# Transposable elements in a marginal plant population: temporal fluctuations provide new insights into genome evolution of wild diploid wheat

**DOI:** 10.1186/1759-8753-1-6

**Published:** 2010-02-01

**Authors:** Alexander Belyayev, Ruslan Kalendar, Leonid Brodsky, Eviatar Nevo, Alan H Schulman, Olga Raskina

**Affiliations:** 1 Institute of Evolution, University of Haifa, Mount Carmel, Haifa, Israel; 2 MTT/BI Plant Genomics Laboratory, Institute of Biotechnology, Viikki Biocenter, University of Helsinki, Helsinki, Finland; 3 Plant Genomics, Biotechnology and Food Research, MTT Agrifood Research, Jokioinen, Finland

## Abstract

**Background:**

How new forms arise in nature has engaged evolutionary biologists since Darwin's seminal treatise on the origin of species. Transposable elements (TEs) may be among the most important internal sources for intraspecific variability. Thus, we aimed to explore the temporal dynamics of several TEs in individual genotypes from a small, marginal population of *Aegilops speltoides*. A diploid cross-pollinated grass species, it is a wild relative of the various wheat species known for their large genome sizes contributed by an extraordinary number of TEs, particularly long terminal repeat (LTR) retrotransposons. The population is characterized by high heteromorphy and possesses a wide spectrum of chromosomal abnormalities including supernumerary chromosomes, heterozygosity for translocations, and variability in the chromosomal position or number of 45S and 5S ribosomal DNA (rDNA) sites. We propose that variability on the morphological and chromosomal levels may be linked to variability at the molecular level and particularly in TE proliferation.

**Results:**

Significant temporal fluctuation in the copy number of TEs was detected when processes that take place in small, marginal populations were simulated. It is known that under critical external conditions, outcrossing plants very often transit to self-pollination. Thus, three morphologically different genotypes with chromosomal aberrations were taken from a wild population of *Ae. speltoides*, and the dynamics of the TE complex traced through three rounds of selfing. It was discovered that: (i) various families of TEs vary tremendously in copy number between individuals from the same population and the selfed progenies; (ii) the fluctuations in copy number are TE-family specific; (iii) there is a great difference in TE copy number expansion or contraction between gametophytes and sporophytes; and (iv) a small percentage of TEs that increase in copy number can actually insert at novel locations and could serve as a *bona fide *mutagen.

**Conclusions:**

We hypothesize that TE dynamics could promote or intensify morphological and karyotypical changes, some of which may be potentially important for the process of microevolution, and allow species with plastic genomes to survive as new forms or even species in times of rapid climatic change.

## Background

Populations are generally viewed as the elementary evolutionary unit [1,2]. A population exists as an integration of individuals in time and space that can vary over a set of successive generations. Under the influence of spontaneous mutations, each population becomes heterogeneous in its genetic structure over time. Thus, a population comprises a mix of different genotypes even if its individuals are more or less phenotypically similar. Under intensive pressure from a particular (usually environmental) factor, a common phenomenon in marginal populations, a shift in the genotypic structure of the population can occur. Such a shift may be regarded as the elementary event of microevolution.

Transposable elements (TEs) may be among the most important internal sources for genotypic population change as a result of their ability to create mutations, alter gene expression, and promote chromosomal aberrations [3-5]. These ancient, ubiquitous, and dynamic components of eukaryotic genomes comprise up to 80% of the large genomes of cereals [6,7]. Data accumulated over the last 30 years suggest that TEs can have major effects on genome organization and function [4,6,8,9]. Nevertheless, it is still unclear whether and to what degree the TEs contribute to evolutionarily significant shifts in the genotypic structure of populations. Population genetic theory assumes that the dynamics of transposable elements in natural populations reflect a balance between the tendency of these elements to increase through transposition and their removal through natural selection acting against individuals with a high element copy number [10,11]. Several studies from different biological systems indicate that the patterns of a single TE family can vary intraspecifically [12-15] and temporally [16]. Examination of the intraspecific dynamics of TEs, especially in marginal populations where microevolutionary processes are intensified [2], could considerably contribute to the understanding of key biological events such as speciation.

One of the most effective ways to understand the dynamics of the TEs over time and space may be an ecological approach, whereby several generations of plants from natural populations are investigated. Here, we have chosen a small, marginal and threatened population of *Aegilops speltoides *(2n = 2× = 14) as a model for exploration of the dynamics of several TE families belonging to Classes I and II (respectively moving via RNA and DNA intermediates). The species is a wild relative of the various wheat species known for their large genome sizes [17,18] contributed to by an extraordinary number of TEs, particularly long terminal repeat (LTR) retrotransposons. *Ae. speltoides *was proposed to be the closest to the wild diploid progenitor of the G genomes and B genomes of polyploid wheat [19-21]. The study population is located on the western banks of the Kishon River (Haifa Bay area, Israel). The population is characterized by high heteromorphy and possesses a wide spectrum of chromosomal abnormalities, including supernumerary chromosomes, heterozygosity for translocations, and variability in the chromosomal position or number of 45S and 5S ribosomal DNA (rDNA) sites [22,23]. We propose that variability in morphological and chromosomal levels may point to variability at the molecular level and particularly in TE proliferation. Thus we aimed to detect and evaluate differences in TE copy numbers between individuals from the Kishon population and to trace inheritance of these deviations in successive generations. The broader goal of the study was to examine whether TE dynamics could be associated with morphological or karyotypical changes, some of which are potentially important for the evolutionary process.

## Results

### Experimental design

*Ae. speltoides *is predominantly a cross-pollinated but self-compatible species [24]. Three original plants were selected from a small (approximately 100 m^2^), marginal, degrade population of *Ae. speltoides *(see Methods). Each selected original genotype represents three groups of previously investigated plants (five to seven individual original spikes in each group), which have been clustered due to their morphological and cytogenetic similarity, including spike morphology, B chromosome existence, appearance of additional 5S rDNA chromosomal clusters, and specific chromosomal rearrangements [22,23].

The progeny from each genotype were obtained in three rounds of selfing. We simulated the situation in nature where, in marginal populations under critical external conditions, outcrossing plants and particularly *Ae. speltoides *very often transit to self-pollination [24-26]. The copy numbers of several TE families were determined by quantitative polymerase chain reaction (qPCR) for each original genotype and its offspring. qPCR data were verified by dot blot analysis. The transpositional activities of TEs were inferred by inter-retrotransposon amplified polymorphism (IRAP) retrotransposon display [27]. Generational changes in the copy number of TE families were compared with those of the non-mobile, highly repetitive tandem repeat *Spelt 52 *and the 5S ribosomal RNA (rRNA) genes (rDNA).

### Dynamics of TE in successive generations

**Figure 1:**
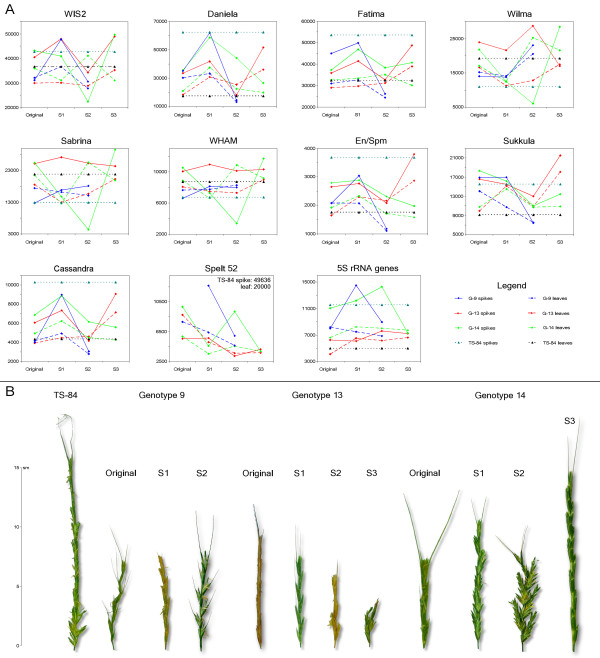
**Copy numbers and morphology**. **(a) **Dynamics of transposable element (TE) copy numbers in three self-pollinated generations of three genotypes from the Kishon population of *Aegilops speltoides *(shown by lines). TE copy numbers of the TS-84 population were used as controls. TE copy numbers for available sibs in selfing generations are shown by separate dots. **(b) **Changes in spike morphology in three self-pollinated generations of three genotypes from the Kishon population of *Ae. speltoides*. Spike morphology of plants from the TS-84 population was used as the control.

The copy numbers of TE families that we investigated are presented in Additional file 1 and in graphic form in Figure 1a. The changes in TE copy number in a set of successive generations was measured by two different methods: qPCR and dot blot. These produced consistent results.

The first salient point is that generative tissues, in most cases, possess higher numbers of TEs than do vegetative tissues. This observation is true for most TEs except those related to the *Athila *family (*Sabrina *and *Wham*). For example, the number of *WIS2 *retrotransposons in the spikes of G13 S1 exceeded that in leaves by 59%, or approximately 18,000 copies. The copy numbers of both the tribe-specific *Spelt 52 *tandem repeats and the 5S rRNA genes were also generally higher in spikes than in leaves (Additional file 1, Figure 1a).

The second remarkable feature is that the copy numbers of all investigated TEs varied significantly over three successive generations, and that each genotype possessed individual TE dynamics over this period. The TE copy numbers decreased or increased significantly, with a rise in one generation followed by a drop in the next and vice versa in an oscillatory fashion. In generative tissues, these oscillations displayed much higher amplitudes. Particular TEs decreased up to 23% from their initial quantity, as in the case of *Daniela *retrotransposons in G9 spikes where the copy number of this element dropped from approximately 62,000 copies in S1 to approximately 15,000 copies in S2 (Additional file 1, Figure 1a). We could not trace further TE dynamics in this genotype since the S2 generation was sterile. The largest absolute rise in TE number was documented also for *Daniela *in G13 spikes where the copy number rose from approximately 18,000 copies in S2 to approximately 52,000 copies in S3. The largest relative rise in TE abundance in one generation was seen for the *Sabrina *element, where in G14 its number in spikes increased by 672% in S3 as compared to S2. A similar jump in this genotype between S2 and S3 was observed also for *WIS2*, *Wilma*, and *Wham*.

Genotypes 9 and 13 were distinct by the amplitudes in their numbers of copies for particular TE families over the generations analyzed, but the tendencies were the same. Common trends included a significant increase of the highly abundant retrotransposons, *WIS2*, *Daniela*, and *Fatima*, in the generative tissues of the S1 generation, followed by a similar decrease in these elements in the S2 generation (Additional file 1, Figure 1a). *En/Spm*-like transposons, *Cassandra *retrotransposons, and 5S rRNA genes exhibited similar dynamics in these genotypes. *Wilma *retrotransposons did not change in the S1 generation but then increased in number in S2. The copy number of *Sukkula *elements did not change in the S1 generation, but then dropped in S2. *Sabrina *and *Wham *elements remained approximately on the same level as in the original plants. The TE dynamics in vegetative tissues were approximately the same as in generative tissues but with less amplitude. For technical reasons, we do not have measurements of the copy number of *Spelt 52 *in the spikes of the original plants of G9, but the rest of the measurements showed a tendency for this tandem repeat to decrease in abundance in generative and vegetative tissues over the generations examined.

Genotype 14 differed in TE copy number temporal dynamics from the two previously described genotypes. Cytogenetic data (see below) make it possible to propose that this genotype was already self-pollinated for at least one generation. *WIS2, Wilma, Sabrina, Wham*, and *Sukkula *elements demonstrated significant, successive decreases in copy numbers in S1 and S2 that were then followed by increases in S3. *Daniela, En/Spm*, and *Cassandra *elements increased in copy number in S1 and successively decreased in S2 and S3. *Fatima *retrotransposons exhibited copy number dynamics in G14 similar to those seen in G9 and G13: increased abundance in S1, decreasing in S2, and again increasing in S3. In G14, too, the TE dynamics in vegetative tissues were approximately the same as for generative tissues, but with less amplitude. The number of *Spelt 52 *repeats, both in spikes and leaves, decreased in S1, increased in S2, and decreased in S3. In spite of a significant increase of copy number in S2, the overall tendency was for reduction. The 5S rRNA genes successively increased in copy number in S1 and S2, and then decreased in S3.

To explore segregation in the progenies of a single genotype, TE copy numbers in sibs from G9 and G13 genotypes were determined (Table S1 in Additional file 2). Significant variations over three generations of two genotypes were observed. The amplitude of TE abundance in sibs could not be explained by simple chromosome segregation (see below) and, therefore, points to mobile element activity.

**Figure 2:**
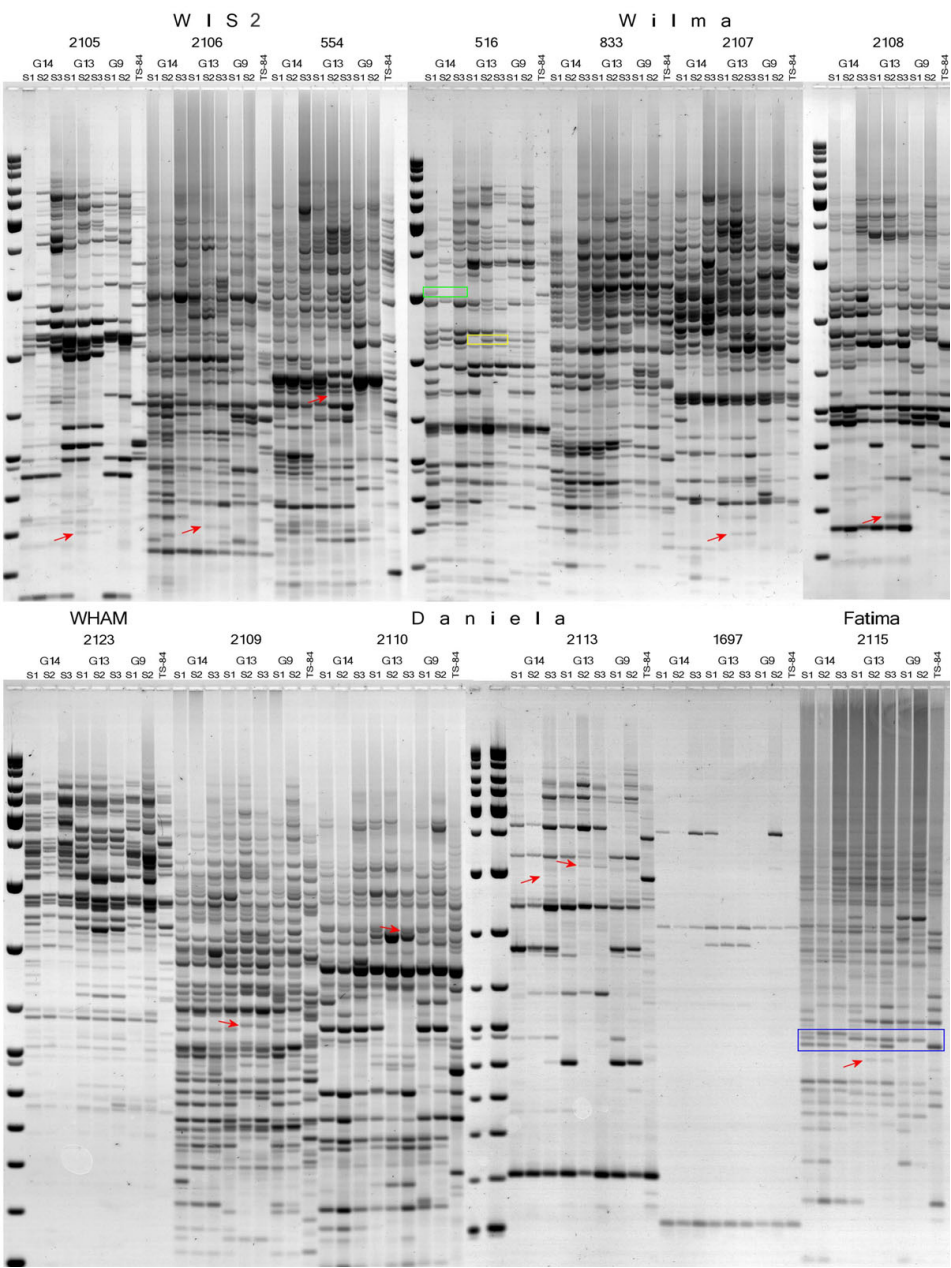
**Inter-retrotransposon amplified polymorphism (IRAP) analyses for several transposable elements (TEs) in the progeny of three genotypes**. Unique bands that appear in S2 and are inherited in S3 are shown by red arrows. An example of heterozygosity displayed in the IRAP pattern is shown in the blue square. An example of band loss in S2 and S3 is shown in the green square. An example of band appearance in S2 and S3 is shown in the yellow square.

A TE display method such as IRAP [27] would be expected to show polymorphisms consonant with large changes in TE copy number. Here, IRAP analyses were conducted on DNA from spikes at the microsporogenesis stage, and showed a high level of polymorphism from generation to generation (Figure 2). G13 showed the most unique bands, with retrotransposons *WIS2 *and *Daniela *producing the greatest number.

**Figure 3:**
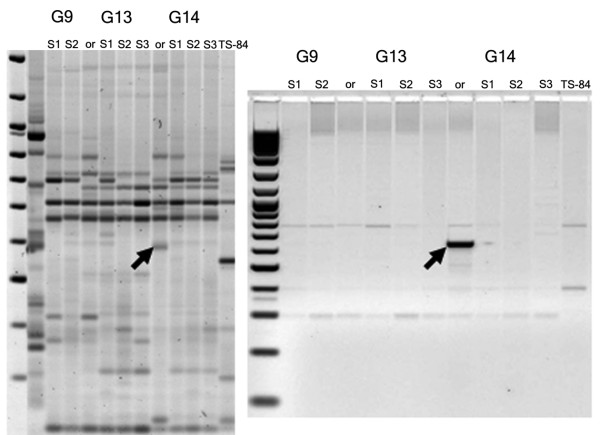
**Analysis of unique inter-retrotransposon amplified polymorphism (IRAP) bands**. In this example, IRAP was conducted with primer 2109, matching the *Daniela *retrotransposon long terminal repeat (LTR) (Table S2 in Additional file 2). The band was cut and sequenced. Two new primers were designed to match the sequence and the uniqueness of the band was checked on the set of DNA samples. This new insertion is in repetitive DNA (Table S2 in Additional file 2).

Various forms of recombination may, of course, bring TEs sufficiently close that a new IRAP band would appear in the absence of a new integration event. For any given IRAP polymorphism, its origin as an integration event can be explicitly proven only by identifying an empty site, from a plant line missing the IRAP product, for one of the two TEs that together served as the template. To find evidence for individual transformation events, we cloned and sequenced 20 unique IRAP bands (Figure 3). At least half of the new insertion sites were in repetitive DNA, and therefore unsuited for identifying the original empty sites. Of the 20, (Table S2 in Additional file 2), 4 of them (a *Daniela*, a *Wis2*, and 2 *Sukkula *insertions) were in non-repetitive sites that could be identified in a sequence database. The advent of new, unique, amplified IRAP bands that resulted from new TE insertions confirms the existence of transpositionally active TEs in the three genotypes of *Ae. speltoides*.

### Multivariate analyses

**Figure 4:**
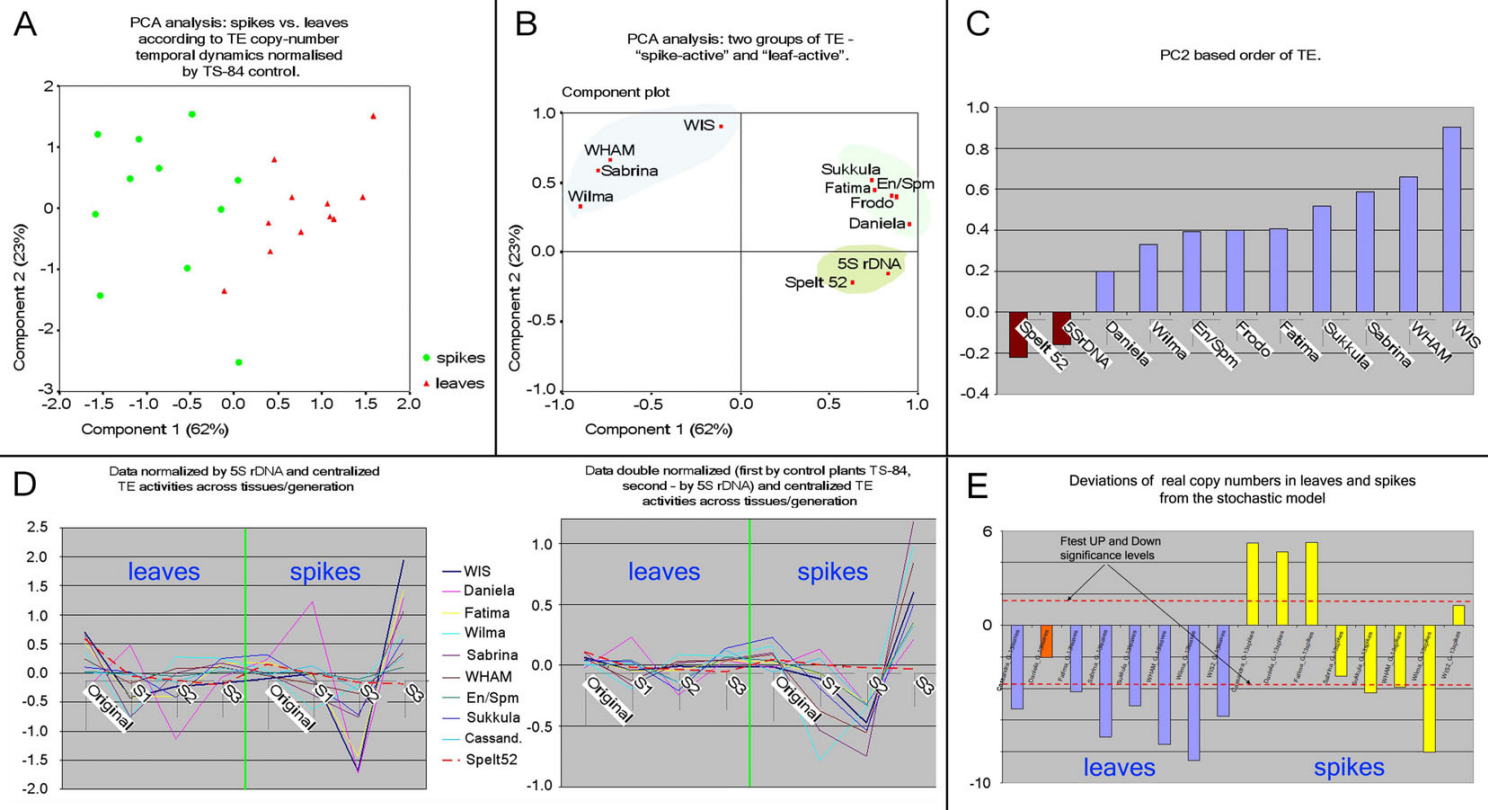
**Principle component analysis (PCA)**. **(a) **PCA analysis: spikes versus leaves for transposable element (TE) copy number changes over the generations, normalized by the TS-84 control. **(b) **PCA analysis: two groups of TE, spike active and leaf active. **(c) **Second principal component (PC2)-based order of TE activation. **(d) **Normalized and centralized patterns of the three genotypes for *Spelt 52 *and TEs across tissues and generations. **(e) **Deviations of real copy numbers in leaves and spikes from the stochastic model.

The data distribution in principle component analysis (PCA) (Figure 4a) demonstrates the clear separation of leaves and spikes for each generation, normalized pairwise for each TE and non-TE repeat in control plants. The separation is along the first principal component (PC1), which covers 62% of initial data variability. The PC1 (Figure 4b) contrasts a group of TEs (*Sukkula, Fatima, Cassandra, En/Spm*, and *Daniela*) and two non-TE sequences (*Spelt 52 *and 5S rDNA) versus another group of TEs (*Wilma, Sabrina, Wham*, and *WIS2*). Therefore, the patterns of relative generational copy number variation of these two groups of TEs regarding their numbers in control plants are different in spikes and leaves. The first group of TEs and both non-TE sequences show larger fluctuations in leaves over successive generations, and the TEs of the second group show larger changes in spikes.

The second principal component (PC2) represents the variability that is independent of the leaf-spike contrast (Figure 4b). When this component is further dissected by type of repeat (Figure 4c), the tissue-independent variance shows opposite trends for TE and non-TE repeats. This dichotomous trend holds across generations, genotypes, and tissues. We further investigated the nature of this trend for all nine TE families. Both raw copy numbers and those normalized by the control data were thus normalized by the number of one of the non-TE sequences (5S rDNA) and by control plants (TS-84) (Figure 4d). For both types of data normalization, the centralized patterns of change in copy number for the TEs and for *Spelt 52 *over the three genotypes and across tissues and generations demonstrate that: (i) the copy number fluctuation of non-TE sequence *Spelt 52 *with respect to 5S rDNA is insignificant across tissues and generations (broken red line) while fluctuations of TE sequences are high; (ii) the intergenerational pattern of copy number changes for all TEs is highly correlated; (iii) there is little relative TE copy number variation in leaves though it is very strong in spikes. It shows a minimum in the S2 generation, and a maximum in the S3 generation. In sum, the separation of TE and non-TE sequences in PC2 appears related to their different patterns of copy number dynamics across generations in spikes.

### Deviations of real copy numbers in leaves and spikes from the stochastic model

**Figure 5:**
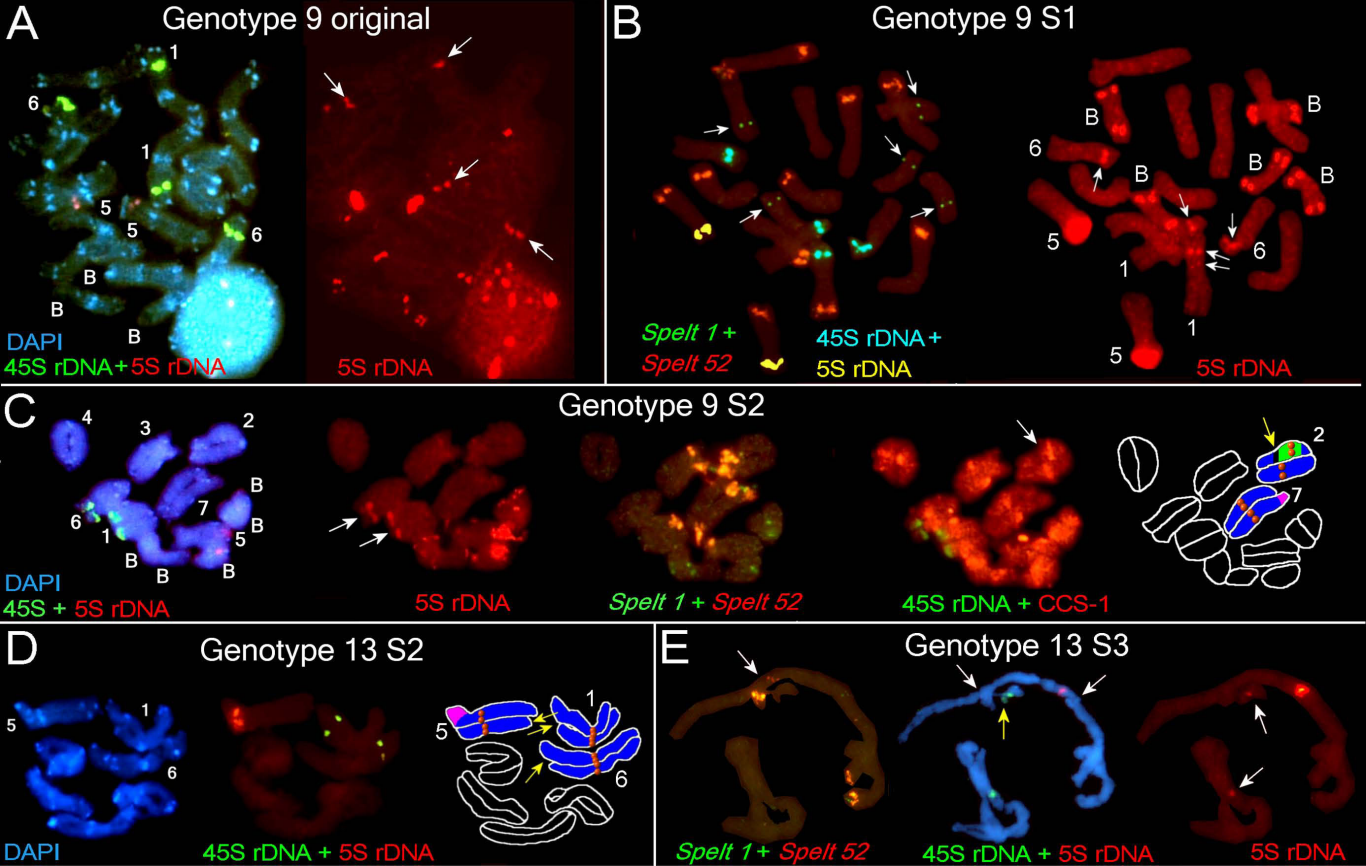
**Fluorescence *in situ *hybridization (FISH) and differential staining with 4',6-diamidino-2-phenylindole (DAPI) on somatic and meiotic chromosomes of *Aegilops speltoides *(part 1)**. **(a) **FISH with 5S rDNA, 45S rDNA and staining with DAPI on somatic chromosomes of the original G9 plant (left). Chromosomes 1, 6 (arrows), and B chromosomes carry additional 5S rDNA sites (right). **(b) **FISH with *Spelt 52, Spelt 1 *(arrows on B chromosomes), 5S rDNA and 45S rDNA on the somatic chromosomes of the G9 S1 plant (left); FISH with 5S rDNA (right). Chromosomes 1 and 6 carry additional 5S rDNA sites (arrowed). **(c) **From left to right: FISH with 5S rDNA and 45S rDNA, and DAPI on the meiotic chromosomes of the G9 S2 plant; 5S rDNA probe alone, chromosomes 1, 6 (arrows), and B chromosomes carry additional 5S rDNA sites; FISH with *Spelt 52 *and *Spelt 1 *on the same chromosomes; FISH with CCS-1 and 45S rDNA (a pericentric inversion is arrowed); the scheme of the main chromosomal rearrangements (see legend). **(d) **DAPI (left) and FISH (middle) with 5S rDNA and 45S rDNA on the meiotic chromosomes of the G13 S2 plant. A scheme of the main chromosomal rearrangements (right). **(e) **FISH with *Spelt 52 *and *Spelt 1 *on the meiotic chromosomes of the G13 S3 plant (left). Small *Spelt 52 *cluster (arrow) marks paracentric inversion in the long arm of the chromosome 5. FISH with 5S rDNA and 45S rDNA with DAPI staining (middle). Both termini of chromosome 5 are involved in heterologous synapses (white arrows); heterozygous deletion on the chromosome 6 is shown by yellow arrow. Chromosomes 1 and 6 carry additional 5S rDNA clusters (arrows in right). The DNA probes and staining: **(a-e) **5S rDNA, *Spelt 52 *and cereal centromere-specific sequence 1 (CCS-1) in red; 45S rDNA and *Spelt 1 *in green; differential staining with DAPI in blue; **(b) **5S rDNA (yellow) and 45S rDNA (blue) in pseudocolors.

**Figure 6:**
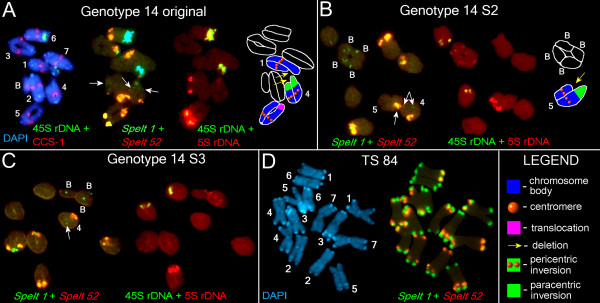
**Fluorescence *in situ *hybridization (FISH) and differential staining with 4',6-diamidino-2-phenylindole (DAPI) on somatic and meiotic chromosomes of *Aegilops speltoides *(part 2)**. **(a) **From left to right: DAPI and FISH with the CCS-1 and 45S rDNA on the meiotic chromosomes of the original G14 plant; FISH with *Spelt 52 *and *Spelt 1*. The clusters of *Spelt 1 *that mark a homozygous paracentric inversion on chromosome 4, and a *Spelt 1 *cluster on the B chromosome are arrowed. FISH with 5S rDNA and 45S rDNA on the same chromosomes. A scheme of the main chromosomal rearrangements. **(b) **FISH with *Spelt 52 *and *Spelt 1 *on the meiotic chromosomes of the G14 S2 plant (left). The clusters of *Spelt 1 *that mark a homozygous paracentric inversion on chromosome 4 and cluster of *Spelt 52 *that marks a heterozygous inversion on the chromosome 5 are arrowed. FISH with 5S rDNA and 45S rDNA (middle). B chromosomes carry 5S rDNA clusters in both arms. The scheme of the main chromosomal rearrangements (right). **(c) **FISH with *Spelt 52 *and *Spelt 1 *on the meiotic chromosomes of the G14 S3 plant (left). Homozygous paracentric inversion on chromosome 4 is arrowed. FISH with 5S rDNA and 45S rDNA on the same chromosomes (right). **(d) **Somatic chromosomes of TS 84, staining with DAPI (left). FISH with *Spelt 52 *and *Spelt 1 *on the same chromosomes (right). The DNA probes and staining: **(a-d) **5S rDNA, *Spelt 52 *and cereal centromere-specific sequence 1 (CCS-1) in red; 45S rDNA and *Spelt 1 *in green; differential staining with DAPI in blue; **(a) **5S rDNA (yellow) and 45S rDNA (blue) in pseudocolors.

The original plants of cross-pollinated *Ae. speltoides *are heterozygous for repetitive DNA sequences, chromosomal patterns, and chromosomal rearrangements (Figures 5 and 6). In order to separate the role of the pre-existing heterozygosity on the TE copy number in the original plants from other mechanisms that can change TE amounts in successive generations, such as transposition, excision, or recombinational elimination, we created a 'naïve' model in which the segregation of parental chromosomes in selfed progenies was simulated. The null hypothesis implies that: (i) cross-pollinated parental plants were initially heterozygous for the content of TEs in the homologues; (ii) only the random segregation of homologues in meiosis during male and female gametogenesis leads to a change in TE copy number in inbred offspring. For modeling we took only the G13 genotype as the genotype with a stable chromosome number, and only the dynamics of Class I elements were simulated.

We carried out 100 realizations for stochastic time course simulations of copy numbers in spikes and leaves across ten generations. Thus, the copy numbers in leaves and spikes were calculated in 100 simulated siblings of each generation of plants, and these 100 simulated siblings from each of the first three plant generations form the generation-specific basic distributions of copy numbers in leaves and spikes. The deviations of copy numbers in real siblings from the basic simulated distributions, separately in leaves and spikes, test the null hypothesis.

The significance of deviations in each generation was calculated by a *t*-test and the non-parametric Mann-Whitney U test (Table S7 in Additional file 2). The significance results of two tests are in good mutual concordance. Taking this into consideration, the F-test statistics were used for significance estimation of the cumulative deviation in positive or negative directions of real data from simulated siblings across three generations for each type of TE separately. The F statistic is:

where *W*_max _and *W*_min _are either *W*_p _or *W*_n _depending on which one is larger. The statistic *W*_p _is defined as the sum of squares either of real *t-*test values, if *t-*test is positive, or alternative expected positive *t-*tests (a small value for Q25 left quantile of *t*-test value distribution), if the real *t*-test is negative:

Similarly:

Statistics *W*_p _and *W*_n _are chi-square distributed with degrees of freedom equaling three each. Thus, the F-test has (3,3) degrees of freedom. The test shows how corroborative *t*-test directions and values are across three plant generations for the particular TE type. The + and - log *P *values of this F-test statistic across TE types are shown in Figure 4e. The sign of the log *P *value reflects the voting of *t*-tests either up (*W*_max _= *W*_p_; *W*_min _= *W*_n_) or down (*W*_max _= *W*_n_; *W*_min _= *W*_p_) from simulations. Thus, long down bars indicate significant cumulative deviation of real data down from simulations across three plant generations.

The graph (Figure 4e) shows that copy numbers of all TE types besides Daniela in leaves of G13 were significantly downregulated in relation to the simulations, but this is not true for spikes. The explanation could be that a high TE copy number in the male gametes of the original plant infers a generally high simulated level of copy numbers in spikes and leaves. However, a plant could survive only if the TE copy number is relatively low in diploid cells, permitting, however, a high copy number in gametes. Thus, plants with high copy numbers, both in gametes and diploid cells, will be eliminated from the population, providing the observed difference between real copy numbers in gametes and in diploid cells for survivors.

### Morphological and karyotypical changes in successive generations

*Ae. speltoides *is a dimorphic species appearing in natural populations as two morphotypes or subspecies;*Ae. speltoides *ssp. *ligustica *and *Ae. speltoides *ssp. *aucheri*. The *aucheri *type is characterized by cylindrical spikes with widely interspersed spikelets. The apical spikelet is awned, and the rachis is tough. The *ligustica *type is characterized by denser, two-rowed ears in which the lateral spikelets are also awned and the rachis is brittle (Figure 1b). Significant morphological and karyotypic abnormalities in successive generations were observed, particularly: reduction of spike awns, appearance of intermediate *ligustica-aucheri *phenotypes, abnormal quantity of spikelets and florets, and spike size reduction (Figure 1b).

For karyotypic analysis we used several chromosomal markers in fluorescent *in situ *hybridization (FISH) experiments: 5S rDNA and 45S rDNA probes, cereal centromere-specific sequence 1 (CCS-1) [28], species-specific *Spelt 1 *[29] and tribe-specific *Spelt 52 *[30] tandem repeats. These probes were used for chromosome identification and localization of chromosomal rearrangements. The genomic formulas for the investigated plants were as follows: G9 (2n = 14 + 3B), G9 S1 to S2 (2n = 14 + 5B), G13, S1 to S3 (2n = 14), G14 (2n = 14 + 2B), G14 S1 (2n = 14 + 3B), G14 S2 (2n = 14 + 4B), and G14 S3 (2n = 14 + 3B) (Figures 5 and 6).

### Dynamics of Spelt 1, Spelt 52, and 5S rDNA clusters in successive generations revealed by FISH

Cytogenetical analysis revealed dramatic differences between genome organizations of plants from the Kishon population in comparison with TS-84 plants representing the center of the *Ae. speltoides *distribution. First, Kishon plants lost a majority of species-specific tandem repeat *Spelt 1*, which appears usually as a component of terminal adenine and thymine (AT) -rich heterochromatic blocks in both arms of almost all chromosomes in TS-84 (Figure 6d). Only one or two terminal clusters of *Spelt 1 *per diploid genome were revealed in all studied genotypes, which means that up to 97% of the clusters were eliminated (Figure 5b, c, e and Figure 6c, Table 1). We observed a similar picture for *Spelt 52 *distal clusters: 57% to 82% were eliminated in genotypes from the Kishon population (Figures 5 and 6, Table 1). Thus, the amount of *Spelt 1 *is 13 times lower and *Spelt 52 *1.5 times lower than in the control genotype TS-84 from the center of the species distribution. These data show that in the marginal Kishon population a high ratio of recombination occurs in terminal and distal chromosomal regions. Evidently, terminal clusters of *Spelt 1 *are the primary targets for deletions, followed by distally located *Spelt 52 *clusters. To the extent that chromosome termini have a similar structure (AT-rich heterochromatic bands enriched with clusters of *Spelt 1 *and/or *Spelt 52 *tandem repeats and TEs), they could be involved not only in homologous but also in heterologous recombination followed by chromosomal rearrangements. In the Kishon population we observed an irreversible elimination of terminal, species-specific *Spelt 1 *tandem repeats and a significant reduction of the distal, tribe-specific tandem repeat *Spelt 52 *as a result of high rates of homologous and heterologous recombination in this small inbred population.

**Table 1:** Number of *Spelt 1, Spelt 52*, and 5S ribosomal DNA (rDNA) clusters and percentage of major chromosomal rearrangements per diploid genome in studied genotypes

Genotype	Spelt 1		Spelt 52		Inversions (paracentric and pericentric)		Translocations		B chromosomes	5S rDNA (chromosomes 5, 1, 6, B chromosomes)	
	
	Sum	Deletions terminal %	Sum	Deletions distal %	Sum	%	Sum	%	Sum	Sum	%
Norm*	28	0	28	0	0	0	0	0	0	2	100
TS 84	26	7.14	18	35.71	2	7.14	0	0	0	2	100
G9 original	2	92.86	9	67.86	0	0	2	7.14	3	12	600
G9 S1	2	92.86	11	60.71	0	0	1	3.57	5	16	800
G9 S2	2	92.86	9	67.86	2	7.14	1	3.57	5	16	800
G13 original	2	92.86	12	57.14	1	3.57	2	7.14	0	2	100
G13 S1	1	96.43	12	57.14	0	0	2	7.14	0	2	100
G13 S2	2	92.86	6	78.57	2	7.14	2	7.14	0	2	100
G13 S3	1	96.43	6	82.14	2	7.14	2	7.14	0	6	300
G14 original	2	92.86	10	64.29	2	7.14	0	0	2	10	500
G14 S1	2	92.86	8	71.43	0	0	2	7.14	3	12	600
G14 S2	2	92.86	9	67.86	1	3.57	2	7.14	4	14	700
G14 S3	2	92.86	10	64.29	2	7.14	2	7.14	3	12	600

*Norm = each chromosome in diploid genome (2n = 14, 28 chromosome arms) potentially carries 1 *Spelt 1 *and 1 *Spelt 52 *cluster in each arm (that is, 28 clusters in total of *Spelt 1 *equated to 100%, and 28 clusters of *Spelt 52 *equated to 100%). Deficiency of 1 *Spelt 1 *cluster (that is, single terminal deletion per 28 chromosome arms) equated to 3.57%, the same as deficiency of one *Spelt 52 *cluster (single distal deletion) equated to 3.57% per diploid genome.

Inversions and translocations: single inversion or translocation per 28 chromosome arms equated to 3.57%. Markers for inversions: CCS-1, telomeric probe, *Spelt 1, Spelt 52*, and AT-positive heterochromatic bands.

Normally chromosome 5 carries one 5S rDNA cluster (two clusters per diploid genome equated to 100%).

Extranumerary B chromosomes regularly carry one 5S rDNA cluster in each arm, thus a single B chromosome adds two 5S rDNA clusters per diploid genome accordingly.

Chromosomes 1 and 6 could carry additional 5S rDNA clusters in homozygote and heterozygote, consequently each among them could add a single additional 5S rDNA cluster, from one to four clusters per diploid genome.

CCS-1 = cereal centromere-specific sequence 1; rDNA/RNA = ribosomal DNA/RNA.

FISH experiments revealed that a significant increase or decrease in copy number might happen without change in the cluster quantity. For example, we traced a significant reduction in *Spelt 52 *copy number from the G9 original plant to G9 S2 (Figure 1a), while cluster numbers increase from 9 to 11 in S1 and drop again to 9 in S2 (Table 1). In spikes of G14 S2 9,200 copies of *Spelt 52 *are shared between 9 clusters while in S3 only 3,800 copies are assigned to 10 chromosomal clusters (Table 1 and Additional file 1). The dynamics of *Spelt 52 *clusters in G14 are as follows: the G14 original line, 10 clusters; G14 S1 to S8; G14 S2 to S9; and G14 S3 to S10 clusters per diploid genome. However, the chromosomal distribution of *Spelt 52 *clusters significantly differs between these genotypes. Thus, both homologues of chromosome 6 carry *Spelt 52 *clusters in the long arms of the original (Figure 6a) and S1 plants, while no clusters were detected (that is, all clusters have been deleted) as early as in S1 (not shown), S2, and S3 (Figure 6a and 6b). One homologue of chromosome 5 in the G14 original plant carries two clusters in the heterozygote in the long arm, where the second cluster is a result of translocation (Figure 6a). In S1 and S2 only one cluster still exists in the heterozygote, which provides evidence for deletion of a second cluster as a result of, most probably, homologous recombination. Thereafter, in S3, both homologues carry *Spelt 52 *clusters in the long arms, which is in accordance with simple chromosome segregation (Figure 6b, c). Chromosome 4 in the original plant is heterozygous for the *Spelt 52 *cluster due to a deletion in the 4L, while in S1 to S3 both homologues are normal and carry *Spelt 52 *clusters in the long arms, which might have occurred by simple inheritance. Therefore, the number of *Spelt 52 *clusters in successive generations follows chromosomal segregation, while copy number abundance of this tandem repeat in each successive genome is subject to amplification or reduction as a consequence of homologous and/or heterologous recombination in distal or terminal chromosomal regions.

We traced similar tendencies for 5S rDNA. The number of clusters and the copy number are not correlated. The abundance of 5S rDNA jumped from 8,000 in the original plant up to 14,500 copies in G9 S1 spikes and thereafter dropped to 9,000 in S3. At the same time, the number of B chromosomes which carry additional 5S sites, and through which we can propose 5S rDNA copy number change, was three in the G9 original plant and five in S1 and S2 generations. The numbers of additional 5S rDNA sites (four in total) in chromosomes 1 and 6 were constant in G14 successive generations (Table 1 and Additional file 1, Figure 1a); consequently, their impact on total 5S rDNA copy number abundance, including regular 5S rDNA clusters on chromosome 5 could be a function of intracluster amplification or reduction.

The occurrence of additional 5S rDNA clusters on both homologues of chromosomes 1 and 6 in the nucleolar organizing regions (NORs) in the G13 S3 plant requires special discussion (Table 1, Figure 5e). Additional 5S rDNA clusters on chromosomes 1 and 6 and the cluster inheritance and elimination in successive generations were reported previously [22,23]. In contrast to G9 and G14, there were no extranumerary 5S rDNA sites in the G13 original plant, and we selected this genotype as a representative sample having the normal chromosomal number of 2n = 14 (no B chromosomes) and two normal 5S rDNA clusters on chromosome 5. There were no additional 5S rDNA clusters in chromosomes 1 and 6 in the offspring in S1 and S2 (Table 1): thus, 5S rDNA clusters arise in the S3 generation. An insignificant variation in 5S rDNA copy number from S2 to S3 provides evidence for genesis of additional 5S rDNA clusters as a result of simultaneous heterologous recombination involving chromosomes 1, 6, and 5 [22,23]. In the genotypes studied, a number of inversions and translocations were also revealed. Major chromosomal rearrangements are shown in Table 1.

### Comparison of TE dynamics and karyotypic events

Despite the fact that fluctuations in copy number are TE-family specific, we can specify at least one common tendency. The maximum copy number dropped in spikes for a majority of TEs, except *Wilma*, which was detected in the S2 generation (Figure 1a, Additional file 1), strongly correlating with the increase in chromosomal rearrangements and particularly with distal or terminal deletions of *Spelt 52 *clusters and AT-rich heterochromatic blocks (Table 1, Figures 5 and 6). For G14 this peak partly shifted to S1 because the original plant was already self-pollinated for at least one generation. This may be related to a homozygous inversion in chromosome 4 (Figure 6a). Most probably, not only the quantity of chromosomal rearrangements, but also their peculiarities (that is, a pericentric inversion on chromosome 2 in G9 S2 causes semi-sterility; non-reciprocal translocation in chromosome 5, plus two deletions on chromosomes 1 and 6 in G13 S2; or deletion plus inversion in the chromosome 5 in G14 S2, and so on) significantly affect the whole genome.

## Discussion

### TE dynamics over time

When we traced the dynamics of TEs over three rounds of selfing, it was discovered that various families of TEs vary tremendously in copy number between individuals from the same population and the selfed progenies, and that fluctuations in copy number are TE-family specific (Additional file 1, Figures 1a and 4b, c). This was especially true in generative tissues at the stage of male gametogenesis, where a greater than sixfold change in a single generation was seen for *Sabrina *elements, for instance. In vegetative tissues, the maximum increase observed was only twofold (*Wilma *elements). We propose that, more than the differences between tissues, this phenomenon reflects differences between the alternating sporophyte and gametophyte generations.

The gametophyte generation in higher plants is produced following many clonal, somatic divisions, unlike in animals, but in virtually all cases (except plants such as *Kalanchoe pinnata*) the leaf does not contribute to the production of the gametophyte. We carried out principal component analysis (Figure 4a) and normalization of TE copy number by non-TE sequences (5S rDNA) and by the number of TEs in the control population from the center of the species distribution (Figure 4d). These analyses revealed that the abundance of TE families as a group was almost constant across generations in the sporophyte, whereas the TE copy numbers in gametophytes reached a minimum at the S2 generation and a maximum at the S3 generation. The latter correlates with karyotypic change (Figures 5 and 6). Modeling of TE copy number temporal dynamics also showed that, in gametophytes, the observed amount of retroelements in the progeny of a single genotype cannot be accounted for by simple chromosomal inheritance, but requires additional mechanisms (Figure 4e).

Therefore, at least a part of the variation observed in TE abundance can be explained by TE activation and subsequent insertion of new copies and by specific loss of integrated TEs. The IRAP display data, in particular the appearance of unique novel bands, are consistent with TE activation. In IRAP, the insertion of a retrotransposon near another creates a new template for PCR amplification. IRAP data and cloning of the unique IRAP bands make it possible to propose that a small percentage of TEs that increase in copy number are transpositionally active and can actually insert at novel locations serving as a *bona fide *mutagen.

We were unable to estimate TE abundance per individual pollen mother cell or monitor the impact of each chromosomal mutation in the total TE dynamic and morphological changes in individual plants. Moreover, the real pattern of chromosomal abnormalities is likely to be much more complex than we can recognize using a limited number of chromosomal markers (Table 1, Figures 5 and 6). Nevertheless, two kinds of chromosomal mutations that occur simultaneously in the same anther, genome-specific and cell-specific mutations, could be recognized in male gametogenesis. The latter could reach approximately 3% to 8% per anther (that is, per single cytological slide containing 20 to 40 well spread cells appropriated for analysis) [22]. Evidently, selection against deleterious mutations additionally reduces our possibility for analysis: we lose a set of data with a number of lethal genotypes in each inbreed generation.

The surviving plants showed a range of cytological changes, and the observed changes in TE abundance could reflect the loss or gain of chromosomal segments that differ in their complement of TEs. We applied a set of non-TE markers (5S rDNA, *Spelt 1*, and *Spelt 52*) that could be followed both cytologically and by their copy number in order to separate TE activity from chromosomal aberrations and reorganization *per se*. It is very likely that distal or terminal deletions of heterochromatin and intracluster copy number reduction of *Spelt 52 *and 5S rDNA tandem repeats physically causes a total decrease in TE amount. As it was repeatedly shown, different types of transposons are clustered in and around ribosomal sites. TEs form chromosomal miniclusters in euchromatin, and also comprise a huge part of the heterochromatin (for review see [31]). Any TE cluster could be considered as an additional target for homologous and heterologous recombination that may cause full or partial deletions of heterochromatic blocks enriched with TEs [32]. In this way chromosomal rearrangements may affect TE copy numbers. Regarding the extranumerary B chromosomes, we could not separate their affect on TE dynamics from other chromosomal abnormalities [33-36]. Our data set does not show a distinct correlation between an increase in the number of B chromosomes and a simultaneous jump in the TE copy number. The precise TE content of B chromosomes is unknown. In this work we explored only 9 of the TEs from more than 100 that were found for *Ae. speltoides *in the GenBank and Triticeae Repeat (TREP) databases. Nevertheless, a certain correlation could be seen. In G9, for instance, increasing B chromosomes from 3 to 5 correlate with significant increase of copy number of *WIS2, Daniela, Fatima*, and *En/Spm *elements in S2 (Figure 1, Additional file 1). In the next generation repetitive DNAs of B chromosomes serve as additional targets for illegitimate recombination, which is proposed as the main driving force behind genome size decrease [37]. Thus, one event (increase of B chromosomes) induces the other (illegitimate recombination) as a response, and this correlates with a decrease of copy number of the several elements. For G14 these trends are not seen so distinctly, perhaps because B chromosome numbers do not change so significantly.

Plant architecture is very highly multigenic. In *Ae. Speltoides*, differences in spike morphology were proposed to be encoded by tightly linked genes with a dominance of *ligustica *over *aucheri *[38]. Some chromosomal rearrangements may target gene complexes, even if these rearrangements are in the heterozygote. This assumption is supported by very significant changes in spike morphology of G13 S2 and G 14 S2 (Figure 1b). Reduction of spike size, reduction of awns, and duplicated floret numbers prove that the gene complex encoding spike morphology is affected. Evidently, these morphological changes could be re-established in the next generation, as is seen for G14 S3, due to chromosomal segregation and selection against the majority of the deleterious aberrations. In other cases, these disorders could be even more profound in the next generation, as we see in G13 S3 (Figure 1b). The particular loci responsible for the spike morphology variations we discuss have not yet been positionally cloned. Furthermore, there are numerous potential mechanisms by which TE expression or integration might affect the expression of the genes determining plant architecture. These range from insertional activation in promoters or coding regions to readthrough induction of RNA interference (RNAi) effects to epigenetic mechanisms, including TE-targeted methylation islands. What role(s) TEs play in chromosome structure, aside from general correlations between TE abundance and heterochromatization on the one hand, or the role of specialized telomeric TEs in telomere structure on the other hand [39,40] are still unknown. The *Brca1 *gene [41] presents a striking example in humans of how a defective repair mechanism can be associated both with TE activation and with chromosomal aberrations, as well as phenotypic changes; however, the cause and effect relationships among these phenomena, even given a clear etiology, are not trivial.

### Possible mechanisms of TE activation

The data raise two key questions: what induces the mass amplification of TE following selfing, and what are the consequences of this process forsuccessive generations? The TEs, particularly retrotransposons, are well known to be activated by biotic and abiotic stress [42-44]. A reduction in DNA methylation is likewise known to activate TEs [45]. McClintock, furthermore, referred to 'genome shock' as an activator of TEs [46]; the phenomena we have observed in *Aegilops *mirror her descriptions. Taking these strands together, TE may be activated globally through chromatin hypomethylation or more specifically *via *changes in the expression of transcriptional or other interacting cellular factors. The observation that not all TEs were activated in spikes supports the latter interpretation.

### Consequences of TE activation

Fluctuations in TE copy number may reflect propagation without fixation. For a new TE insertion to be heritable, it must occur in a cell clonally giving rise to a germ cell. Of the TE families we examined, *WIS2, Wham, Sabrina*, and *Wilma *were the most spike-active, showing the highest levels of copy number change in the gametophyte, whereas *Fatima, Daniela, Sukkula, Cassandra*, and *En/Spm *showed the greatest fluctuations from generation to generation in leaves. The various steps of the retrotransposon life cycle, from transcription of mRNA to integration of cDNA, are likely involving interactions with cellular proteins. The distinct behavior of these two groups of retrotransposons may reflect differences in these interactions.

If an insertion in a meristematic cell hampers that cell's replication, the cell may be replaced by a neighbor due to the regulative nature of plant meristems. Even if a new TE insertion arises in a germ cell, that cell may not give rise to viable embryos. The drop in TE numbers observed from S1 to S2 spikes could reflect selection against deleterious insertions. Methylation in subsequent generations can, furthermore, silence the activity of TE insertions that are not themselves lethal. Moreover, retrotransposons are removed over time both by LTR-LTR recombination, leading to the generation of solo LTRs [47], and by illegitimate recombination [37]. Ultimately, the fate and fixation of new TE insertions depends on events not only at the level of the nucleus, but also at the population level.

In natural plant populations, TE dynamics may be associated with changes in pollination direction [11,48]. Under critical external conditions, outcrossing plants very often transit to self-pollination [25,26,49,50]. We have observed transitions between cross-pollination and self-pollination not only in *Ae. speltoides*, but also in closely related *Ae. sharonensis *as well as in *Hordeum spontaneum *(unpublished results). We simulated this process by artificially transiting cross-pollinated plants of *Ae. speltoides *to self-pollination in the greenhouse. Inbreeding in plants [51] and *Drosophila *[52] can have diverse physiological and phenotypic consequences, which have been hypothesized to derive from the epigenetic effects of pairing interactions between chromosomes [53]. Epigenetic changes may in turn lead to TE activation, which is associated with high rates of karyotypic change [6], genetic variation (though limited in marginal populations) and epigenetic alteration, an important source for phenotypic variability [54]. Some of these mutations could be heritable [22,23,55]. It is therefore tempting to hypothesize that the TE activation we observed might be a primary or secondary effect of the mating system change, though substantiating evidence is still lacking.

Theory predicts that it is difficult for a genetic variant to become fixed except in small, inbred populations [56]. Population size reduction increases the probability of fixation of deleterious mutations [57] and also leads to genome diversification via the accumulation of molecular changes [58]. Moreover, smaller population sizes may facilitate the passive accumulation of TE insertions [59]. From 2000 to 2005, the population under study here decreased threefold in size. It is also essential to note that this population is located on the southern edge of the species range, where it is under abiotic stress. These stresses, therefore, may simultaneously activate TEs directly, increase self-fertilization and thereby set off the phenomena we observed here, and limit population size, increasing the likelihood of the fixation of genomic changes. The combination of genetic and epigenetic alterations with karyotypic change allows species with plastic genomes to survive as new forms or possibly species under intensive environmental pressure.

## Methods

### Plant material and DNA extraction

Individual spikes of cross-pollinated *Ae. speltoides *(2n = 14) were collected in the year 2000 from the Kishon populations. The population is small (approximately 100 m^2^) and represents the southern extent of the species range. *Ae. speltoides *is a dimorphic species (ssp. *aucheri *and ssp. *ligustica*) (Figure 1b), and the differences in spike morphology are controlled by a block of closely linked genes [24,38]. Analysis of spike size, the morphology of glumes (presence or absence of the keel tooth), and the degree of lateral awn expression (for ssp. *ligustica*) revealed significant intrapopulation morphological variability in *Ae. speltoides*, which guided cytogenetical screening thereafter.

Based on previous cytogenetic data, we selected three original plants of *Ae. speltoides *for further investigation, each from different spikes. Earlier data showed that some siblings can carry chromosomal abnormalities and extranumerary B chromosomes [22,23]. Thus, we expected that extranumerary B chromosomes and other chromosomal rearrangements could appear in the offspring of the chosen maternal genotypes. Phenotypically, genotype 9 (G9) exhibited *ligustica *features, while genotypes 13 (G13) and 14 (G14) were confirmed as the *aucheri *type (Figure 1b).

Young spikes (two to five, depending on individual plant vigor) at the male gametogenesis stage (when spikes are approximately two-thirds to three-quarters of a flag leaf) were selected for meiotic chromosome analysis and, at the same time, for DNA extraction. We used total DNA from two sources, one from young leaves and the other from spikes at the microsporogenesis stage, extracted by the cetyltrimethylammonium bromide (CTAB) method. The DNA showed equal purity and quality from all samples. At the same time, other spikes from the same plants were used for meiotic chromosome analysis, and the rest of the spikes were selfed in order to produce seeds for the next generation. Plants of *Ae. speltoides *ssp. *aucheri *TS-84 (Latakia, Syria), from the center of the species range, were used as controls.

### TE sequence sources and primer design

Sequences of transposable elements were taken from the TREP database (see Table S3 in Additional file 2; [60]). For each TE family, sequence accessions were aligned and conservation assessed with the multiple alignment procedure of MULTALIN [61]. The conserved segments of the LTR or internal domain of the retrotransposons were used for the design of PCR primers, which was carried out with the program FastPCR [62]. We designed several primer pairs for each repeated element or TE to compare the efficiency and reproducibility of amplification. None of the primer pairs that were chosen formed dimers and all show high PCR efficiency. The chosen primers match motifs sufficiently conserved to allow amplification of almost all TE family members in the genome.

### PCR and IRAP methods

For molecular genetic procedures we used standard protocols. IRAP analysis was made according to Kalendar and Schulman [27]. Protocol details and primer sets for quantitative real-time PCR, long-range PCR, dot blot hybridization and analysis, and IRAP can be found in the Tables S4-S6 in Additional file 2, and in Supplemental methods in Additional file 2. A total of 20 unique IRAP bands were cloned and sequenced (see Table S2 in Additional file 2, Figures 2 and 3).

### Data normalization and centralization

We used three types of data normalization (Figure 4). In the first, the temporal dynamics of nine TE and two non-TE sequences (*Spelt 52 *and 5S rDNA) at the S1, S2, and S3 generations were contrasted with the temporal dynamics of the same TE in the control genotype (TS-84). The normalization was tissue and TE-specific: leaves against leaves and spikes against spikes. Thus, the ratio of copy numbers measures the effect of TE activity in the genotype under study regarding activity of the same TE in the control genotype. The second type of data normalization applied to the temporal dynamics of the TE as a group, and served to evaluate the data with respect to one of the non-TE sequences from the same generation and tissue. The third method of normalization applied was a combination of the first two: first by tissue and TE against control plants, and then against non-TE sequences from the same generation and tissue. To centralize a pattern of temporal dynamics for an individual TE, we subtracted the pattern for non-TE sequences from that of the TE at each tissue and generation point.

### Principal component analysis

The temporal pattern of TE copy number by tissue, generation, and genotype was represented by the vector of the normalized copy number for the control genotype over time for the 9 TE and 2 non-TE sequences (11 values; Figure 4a-c). For the other dimension, the pattern of TE copy number dynamics for each TE and non-TE sequence was represented by its 16 control-normalized activities across genotype, generation, and tissue. The 2-dimensional PCA space covers 85% of control normalized data variability and this space has a good representation of the distribution of genotypes, generations, and tissues in 11-dimensional original space (and distribution of 9 TE and 2 non-TE sequences in 16-dimensional dual space).

### The stochastic simulation of TE-specific copy numbers in leaves and spikes across plant generations

The distribution of copy numbers of different TEs in spikes and leaves across generations of the G13 genotype was contrasted with the computationally simulated distribution of TE copy numbers. For each TE type separately, the stochastic model starts from spikes with equal distribution of TEs over fragments of seven paternal and seven maternal chromosomes in prophase I of male and female meiosis, when synapsis and crossover occur. The TE copy number in male gametes (pollen mother cells (PMC)) was estimated according to observed copy numbers in spikes and leaves of the original plant under the assumption that each spike contains 50% of diploid cells (before telophase I) and 50% of haploid male gametes when four daughter cells are formed, each having only one recombinant chromosome of each homologous pair. Thus, the average copy number in spike's male gametes (M1) was calculated as follows:

where S0 is the measured total TEs copy number in spikes of original plant; F0, TE copy number in maternal haplome; M0, in paternal haplome; M1, in male gametes (PMCs).

The measured TE copy number in leaves (L0) is an average of two haploid genomes: maternal (F0) and paternal (M0). Therefore:

As a result:

and, finally M1 is estimated as:

The F1 TEs copy number in female gametes could be estimated as L0 on average because this haplome contains one parent recombinant chromosome set.

The average of F1 and M1 copy numbers is L1, the average copy number in a diploid genome of the first generation (S1 leaves). The S1 TE copy number (in spikes of the S1 generation) would be calculated after estimation of M2, a copy number in male gametes of the second generation. In order to estimate M2 and F2, we simulated generational change. The steps of the generational change stochastic simulation that produces the distribution of TEs over gametes of the second generation are as follows: (1) estimated F1 and M1 copy numbers in male and female haploid genomes are uniformly distributed over fragments of 7 chromosomes (there are 10 fragments per chromosome by default); (2) each pair of homologues are involved in the crossover with some probability (0.1 by default); (3) after segregation of homologues chromosomes and sister chromatids, the randomly selected seven chromosomes will compose a gamete. Since all chromosomes of our model are equal in uniform distribution of TEs over their fragments, the selection is equivalent to selection of 7 × 10 fragments out of 28 × 10 fragments after the stochastic crossover. After determining M2 and F2 copy numbers, S1 and L2 were estimated as above, and S2 plus L3 were estimated after simulation of the next generation. The further generations were simulated according to the same template.

### Chromosome spread preparation, probe labeling, in situ hybridization, detection, and differential staining procedures

For *in situ *hybridization experiments, 2 to 3 cytological slides (that is, 2 to 3 individual anthers) containing 20 to 50 well spread chromosomal plates at the diakinesis or metaphase I stage were used. The rest of the spikes were selfed in order to produce seeds for the next generation. To prevent contamination, these spikes were bagged at least 5 to 7 days before pollination. Chromosome procedures were as described previously [22,23]. Protocol details can be found in the Supplemental methods in Additional file 2 (see also [63]).

## Competing interests

The authors declare that they have no competing interests.

## Authors' contributions

AB, EN, AS and OR conceived and designed the experiments. AB, RK and OR performed the experiments. AB, RK, LB, AS and OR analyzed the data. AB, LB, AS and OR wrote the paper.

## Supplementary Material

Additional file 1 Transposable element (TE) copy numbers in three self-pollinated generations of three genotypes from the Kishon population of *Ae. speltoides*. Click here for file

Additional file 2 **Various**. Supplementary methods. Quantitative real-time polymerase chain reaction (PCR) and relative quantification. Long distance PCR for transposable elements (TEs). Dot blot hybridization and analysis. Inter-retrotransposon amplified polymorphism (IRAP) for TE transposition. Cloning and sequencing of unique IRAP bands. Chromosome spread preparation, probe labeling, in situ hybridization, detection, and differential staining procedures. Table S1. Relative TE copy numbers in sibs of explored genotypes. Table S2. Sequences with new TE insertions. Table S3. Transposable element accessions. Table S4. Specific primers and primer sets for *Aegilops speltoides *TEs, 5S ribosomal RNA (rRNA) genes and *Spelt 52 *tandem repeats. Table S5. Primer combinations for quantitative PCR. Table S6. Primers for IRAP analysis. Table S7. The significance of deviations in each generation via the *t*-test and non-parametric Mann-Whitney U test. Click here for file
