# Supplementary material for: Transposable elements in a marginal plant population: temporal fluctuations provide new insights into genome evolution of wild diploid wheat
Source: Mob DNA. 2010 Feb 1;1:6. doi: 10.1186/1759-8753-1-6 (PMC2836003; doi:10.1186/1759-8753-1-6)
Supplement: Additional file 1 — Transposable element (TE) copy numbers in three self-pollinated generations of three genotypes from the Kishon population of Ae. speltoides. [file 1759-8753-1-6-S1.DOC]

| **Genotype** | **Generation** | **Tissue** | **Transposable element copies/error in percentage** | | | | | | | | | ***Spelt 52* copies/error** | | **5S rRNA copies/**  **error** |
| --- | --- | --- | --- | --- | --- | --- | --- | --- | --- | --- | --- | --- | --- | --- |
|  |  |  | ***WIS2*** | ***Daniela*** | ***Fatima*** | ***Wilma*** | ***Sabrina*** | ***Wham*** | ***En/Spm*** | ***Sukkula*** | ***Cassandra*** |  |  |  |
| G9 | Original | Spikes | 31,200/2.14 | 35,000/3.16 | 45,000/4.12 | 14,000/2.01 | 12,800/5.23 | 6,600/3.62 | 2,100/5.11 | 16,900/5.25 | 4,000/3.14 | No data | 8,000/1.25 | |
|  |  | Leaves | 32,200/4.63 | 30,200/5.43 | 30,900/4.08 | 15,200/5.11 | 17,500/5.69 | 7,700/9.55 | 2,100/2.37 | 14,100/4.41 | 4,200/5.63 | 7,800/2.63 | | 8,200/6.75 |
|  | S1 | Spikes | 47,800/3.56 | 61,900/3.71 | 49,900/1.07 | 13,800/1.35 | 16,900/5.67 | 8,100/0.86 | 3,100/6.48 | 17,000/10.60 | 9,000/6.33 | 12,600/2.90 | | 14,500/1.10 |
|  |  | Leaves | 36,700/3.68 | 33,300/3.67 | 32,600/1.34 | 14,100/3.75 | 16,200/4.44 | 7,800/3.42 | 2,100/3.48 | 10,700/7.71 | 4,900/7.19 | 6,400/13.01 | | 7,500/15.74 |
|  | S2 | Spikes | 30,600/5.03 | 14,500/9.09 | 26,100/6.27 | 23,100/2.34 | 18,100/4.86 | 8,000/5.84 | 1,200/0.91 | 7,400/2.69 | 3,100/6.11 | 5,900/2.47 | | 9,000/9.30 |
|  |  | Leaves | 27,800/7.00 | 13,200/3.57 | 24,400/10.69 | 20,500/8.61 | 15,100/14.05 | 8,300/9.97 | 1,100/10.10 | 7,500/4.18 | 2,800/14.68 | 4,600/3.36 | | 6,900/3.97 |
|  | S3 | Spikes | Lethal | - | - | - | - | - | - | - | - | - | | - |
|  |  | Leaves | Lethal | - | - | - | - | - | - | - | - | - | | - |
| G13 | Original | Spikes | 40,500/3.64 | 33,400/5.10 | 35,900/5.42 | 23,900/11.83 | 25,200/5.93 | 10,100/5.96 | 2,700/3.69 | 16,600/11.23 | 6,100/4.75 | 5,500/4.94 | | 6,300/2.21 |
|  |  | Leaves | 30,000/2.89 | 18,400/4.94 | 29,000/4.36 | 16,600/3.16 | 18,600/1.04 | 8,100/3.39 | 1,700/6.67 | 9,900/3.31 | 3,900/7.33 | 8,700/2.53 | | 4,100/3.24 |
|  | S1 | Spikes | 48,000/5.90 | 41,700/4.07 | 41,400/7.21 | 21,600/2.35 | 27,200/6.33 | 11,000/10.60 | 2,800/6.30 | 15,500/6.58 | 7,300/7.62 | 5,600/5.60 | | 6,100/28.63 |
|  |  | Leaves | 30,100/14.75 | 30,800/14.42 | 29,800/12.82 | 11,300/8.85 | 13,000/3.74 | 7,500/3.99 | 2,300/5.77 | 15,200/5.49 | 4,500/5.64 | 5,100/9.35 | | 6,500/10.43 |
|  | S2 | Spikes | 34,300/2.39 | 18,200/6.81 | 32,700/1.06 | 28,700/3.78 | 25,400/5.39 | 10,100/4.87 | 2,100/1.22 | 13,000/4.26 | 4,200/9.47 | 3,200/5.08 | | 7,600/9.97 |
|  |  | Leaves | 28,900/5.33 | 25,400/3.10 | 31,200/1.40 | 12,800/0.67 | 15,700/7.98 | 7,300/4.12 | 2,200/1.99 | 10,900/6.32 | 4,600/0.12 | 3,600/3.15 | | 6,200/5.43 |
|  | S3 | Spikes | 49,000/3.61 | 51,500/1.44 | 48,700/3.97 | 17,000/6.62 | 24,400/1.81 | 10,300/4.33 | 3,800/0.63 | 21,500/2.74 | 9,100/8.64 | 4,100/3.06 | | 7,200/11.79 |
|  |  | Leaves | 35,300/5.42 | 35,900/1.95 | 39,000/6.94 | 17,500/12.85 | 20,300/7.43 | 9,000/6.89 | 2,900/3.37 | 18,100/8.88 | 7,200/6.05 | 3,700/6.83 | | 6,600/4.08 |
| G14 | Original | Spikes | 43,200/5.46 | 35,800/5.72 | 37,300/6.19 | 21,800/1.81 | 25,500/5.26 | 10,500/3.08 | 2,800/2.50 | 18,300/11.70 | 6,900/6.87 | 9,800/4.08 | | 11,100/3.69 |
|  |  | Leaves | 36,000/5.23 | 21,000/1.64 | 32,600/1.91 | 17,100/1.91 | 21,500/9.09 | 8,900/0.34 | 1,900/3.82 | 10,800/2.66 | 4,900/1.23 | 5,900/2.86 | | 6,600/7.27 |
|  | S1 | Spikes | 41,000/5.07 | 58,600/9.88 | 46,900/2.53 | 12,500/0.96 | 14,800/0.13 | 7,300/3.51 | 2,900/3.38 | 16,200/9.48 | 8,900/4.29 | 4,600/9.67 | | 12,200/2.07 |
|  |  | Leaves | 31,200/4.59 | 37,400/8.11 | 33,300/5.81 | 12,400/8.09 | 12,600/12.43 | 6,600/11.97 | 2,300/7.64 | 14,500/6.19 | 6,200/8.61 | 3,500/9.68 | | 8,300/4.02 |
|  | S2 | Spikes | 22,500/6.79 | 44,200/9.25 | 38,400/1.46 | 6,100/5.27 | 4,400/5.79 | 3,400/1.33 | 2,300/2.86 | 11,000/5.14 | 6,200/6.14 | 9,200/1.48 | | 14,300/3.18 |
|  |  | Leaves | 41,100/9.79 | 22,500/1.89 | 35,100/5.60 | 25,200/4.93 | 25,600/9.06 | 10,900/6.13 | 1,700/2.71 | 10,700/6.29 | 4,500/2.84 | 4,500/1.55 | | 8,000/5.80 |
|  | S3 | Spikes | 49,700/9.18 | 26,600/3.28 | 40,700/1.60 | 28,400/6.62 | 29,600/2.82 | 11,700/5.35 | 2,000/3.64 | 13,500/8.25 | 5,600/2.55 | 3,800/15.91 | | 7,300/6.38 |
|  |  | Leaves | 31,100/6.90 | 19,900/6.49 | 30,100/4.17 | 21,600/12.85 | 19,900/14.04 | 9,200/2.10 | 1,600/7.67 | 10,900/5.27 | 4,300/7.78 | 3,900/9.43 | | 7,700/12.41 |
| TS-84 | Control | Spikes | 42,800/2.05 | 62,300/2.97 | 53,600/0.76 | 11,000/2.23 | 12,900/8.50 | 6,800/2.69 | 3,700/3.28 | 15,500/3.50 | 10,300/1.48 | 49,600/3.97 | | 11,600/2.71 |
|  |  | Leaves | 36,700/8.72 | 17,500/4.46 | 32,500/4.42 | 19,300/1.29 | 21,900/5.68 | 8,700/5.06 | 1,800/0.35 | 9,200/1.38 | 4,400/2.15 | 20,000/1.00 | | 5,000/2.00 |
| Average error in percentage | | | 5.57 | 5.10 | 4.74 | 4.97 | 6.35 | 4.46 | 4.00 | 5.98 | 5.69 | 5.42 | | 6.81 |

rRNA=ribosomal RNA.
