# Supplementary material for: Transposable elements in a marginal plant population: temporal fluctuations provide new insights into genome evolution of wild diploid wheat
Source: Mob DNA. 2010 Feb 1;1:6. doi: 10.1186/1759-8753-1-6 (PMC2836003; doi:10.1186/1759-8753-1-6)
Supplement: Additional file 2 — Various. Supplementary methods. Quantitative real-time polymerase chain reaction (PCR) and relative quantification. Long distance PCR for transposable elements (TEs). Dot blot hybridization and analysis. Inter-retrotransposon amplified polymorphism (IRAP) for TE transposition. Cloning and sequencing of unique IRAP bands. Chromosome spread preparation, probe labeling, in situ hybridization, detection, and differential staining procedures. Table S1. Relative TE copy numbers in sibs of explored genotypes. Table S2. Sequences with new TE insertions. Table S3. Transposable element accessions. Table S4. Specific primers and primer sets for Aegilops speltoides TEs, 5S ribosomal RNA (rRNA) genes and Spelt 52 tandem repeats. Table S5. Primer combinations for quantitative PCR. Table S6. Primers for IRAP analysis. Table S7. The significance of deviations in each generation via the t-test and non-parametric Mann-Whitney U test. [file 1759-8753-1-6-S2.DOC]

# Additional file 1

**Methods**

## Quantitative real-time PCR and relative quantification.

The 25μl reaction volume contained: 1 × buffer (10mM Tris-HCl, pH 8.8, 50mM KCl, 4mM MgCl_2_, 0.1 % Triton X-100), 25ng DNA, 300nM each primer, 200μM dNTP, 1U DyNAzyme II DNA Polymerase (Finnzymes), 0.5 × SYBR Green I (Cambrex Bio Science Rockland, Inc.), 1 × ROX (6-carboxy-X-rhodamine, Sigma-Aldrich’s reference dye: R4526, 100 × stock) or 1µM 6-ROX. A 10 000 × stock solution of SYBR Green I was used. The 10 000 concentrate was diluted to 1/50 in 50% DMSO in water. Quantitative PCR was performed in an ABI PRISM® 7000 Sequence Detection System (Applied Biosystems) in 96-well plates with an ABI PRISM optical adhesive cover (or “Abgene” 96-well plate AB-1100 and AB-1170 optical clear adhesive cover). The amplification program consisted of: 95°C, 5 min; 25 cycles of 15 sec at 95°C, 60 sec at 60°C, 60-120 sec. at 68°C (fluorescence was monitored in this step). The ABI Prism 7000 SDS Software (provided with the ABI Prism 7000) was used to quantify differences in copy number. Relative quantification compares the cycle threshold (Ct) of unknown samples against a standard curve of a sample with known copy numbers. All DNA samples were repeated three or four times per 96-well plate. A standard curve was developed by plotting the logarithm of known concentrations (2-fold dilution series from 12.5 ng per 25μl reaction volume solution) of the reference sample *Ae. speltoides* DNA (TS-84), in which concentration was determined spectrophotometrically after RNase treatment and purification, against the cycle threshold (Ct) value. The Ct value is inversely proportional to the log of the initial concentration, so that the lower the Ct value, the higher the initial copy number of the TE. The Ct values were automatically selected on the ABI PRISM 7000 for each assay type and the data were exported into Microsoft Excel for further analysis. Amplification efficiency of the reaction is an important consideration when performing relative quantification.

The efficacy of the PCR was determined by recording a standard curve using sequential dilutions of the *Ae. speltoides* DNA (No. 6 - TS-84). A standard curve with a correlation coefficient of about 0.99 and a slope of about −3.3 on a semi-logarithmic plot (a tenfold different concentration of the target gene should result in Ct values with a difference of 3.3) was sought. The efficiency of qPCR and reproducibility of the results did not depend on the length of amplified fragments. Primer combinations for quantitative PCR are shown in the Supplemental Table 3. In practice, correlation coefficient was close to ideal (0.97 to 0.99) for all primer combinations that were used in qPCR.

## Long distance PCR for TEs

The 100μl reaction volume contained: 1 × Phusion HF buffer, 100ng DNA, 300nM each primer, 200μM dNTP, 2U Phusion DNA Polymerase (Finnzymes). The reaction cycle consisted of: 98°C, 30 sec; 15-17 cycles of 10 sec at 98°C, 40 sec at 66°C, and 180 sec at 72°; a final extension of 5 minutes at 72°C. PCR products were purified from the reaction and used as probed in dot-blot analysis.

## Dot-blot hybridization and analysis.

The purified PCR fragment was labeled using [α-32P] dCTP (3000 Ci/mmol, Amersham Biosciences) by the random-primed method (RediPrime II kit, Amersham) and purified by ethanol precipitation. Total DNA was denatured with 0.2M NaOH by incubating for 15 minutes at 37°C, then spotted (25ng per dot) directly onto a Hybond N+ nylon membrane (Amersham Biosciences). After UV cross-linking, the membrane was prehybridized at 65°C for at least 3 h in 5 × SSC, 5 × Denhardt's solution, 0.5% SDS, and 200 μg/ml salmon sperm DNA. After prehybridization, the purified and denatured labeled probe (25 ng) was added to the prehybridization buffer and the membrane was hybridized at 65°C for 12 – 18 h. Post-hybridization washes were made twice in 2 × SSC, 0.1% SDS at room temperature for 10 min, and twice at 65°C for 20 min and then once in 0.1 × SSC, 0.1% SDS at 65°C for 60 min. The membrane was exposed to a phosphorus screen for 2–5 h, scanned with an FLA5100 imaging system (Fuji Photo Film (Europe) GmbH., Germany) scanner at a resolution of 25µm (16-bit per pixel) and analyzed using the AIDA software.

## IRAP for TE transposition.

The PCR was performed in a 25 µl reaction mixture containing 25 ng DNA, 1 × PCR buffer (75 mM Tris-HCl (pH 9.0), 2 mM MgCl_2_, 50 mM KCl, 20 mM (NH_4_)_2_SO_4_, 0.01% Tween 20), 0.2 µM of primer, 0.2 mM dNTPs, and 1 U Biotools DNA polymerase (*Thermus thermophilus* HB27, Biotools S.A.). The PCR program consisted of: 1 cycle at 94°C 4 min; 30 cycles of 94°C for 40 sec, 60°C for 40 sec, 72°C for 2 min; a final extension step of 72°C for 5 min. Amplification was performed in a PTC-100 Programmable Thermal Controller (MJ research Inc., Bio-Rad Laboratories, USA) or a Mastercycler Gradient (Eppendorf AG, Germany) in 0.2 ml tubes or in 96-well plates. Products were analyzed by gel electrophoresis in 1.7 % (w/v) agarose gels (RESolute Wide Range, BIOzym) with 1 × STBE electrophoresis buffer (10 × STBE: 0.2 M Tris-H_3_BO_3_, 0.05 M Na_2_B_4_O_7_, 5 mM EDTA, pH 8.6), at 80V for 7 hours (or 50V for 12 hours) and visualized by staining with ethidium bromide. Gels were scanned on a FLA-5100 imaging system (Fuji Photo Film (Europe) GmbH., Germany) scanner with a resolution of 50µm. (Figure 3).

## Cloning and sequencing of unique IRAP bands.

The unique IRAP bands were extracted (Qiagen MinElute Gel Extraction Kit) from agarose gels (1.7%) after electrophoresis and ethidium bromide staining. PCR fragments were cloned into the pGEM-T vector and sequenced with universal pUC primers. Pairs of new primers were generated based on the obtained sequences and the uniqueness of each individual band was checked on a set of DNA samples (Figure 4**,** left).

## Chromosome spread preparation, probe labeling, in situ hybridization, detection, and differential staining procedures.

In this study, we used several probes for fluorescent *in situ* hybridization (FISH). For *in situ* localization of 45S rRNA and 5S rRNA gene regions, we used the pTa71 and As5SDNAE probes respectively. Probe pTa71 was labeled with biotin-16-dUTP (Roche). The As5SDNAE probe was labeled with Cy-3 (Amersham). Centromeres were localized by hybridization with a PCR-generated probe, CCS-1, labeled with digoxigenin-11-dUTP (Roche). For localization of chromosomal rearrangements and detection of heterochromatin dynamics, the species-specific tandem repeat *Spelt 1* and tribe-specific tandem repeat *Spelt 52* were used. These two sequences have been shown to comprise a considerable part of the heterochromatin of *Ae. speltoides*. Two pairs of primers were designed on the basis of sequences from GenBank (Y09217, AY117400, AY117401, AY117402, Z21644). For *Spelt 1*, the primers were: 5’ TTCTTCTGGCCGTGCCATA, 3’ CTTTTGCCAAATTAGGTACCG. The primers for *Spelt 52* are described above. PCR products were purified and labeled with biotin-16-dUTP (*Spelt 1*) and with Cy-3 (*Spelt 52*). The FISH procedure has been described previously in detail [22]. Following *in situ* hybridization, the same slides were then stained with the AT-specific fluorochrome DAPI. Slides were examined on a Leica DMR microscope using CCD camera DFC300 FX.

# Tables

## Table 1. Relative TE copy numbers in sibs of explored genotypes.

| Genotype | Generation | Plant No | Tissue | Transposable element copy numbers | | | | | | | | |
| --- | --- | --- | --- | --- | --- | --- | --- | --- | --- | --- | --- | --- |
|  |  |  |  | *WIS2* | *Daniela* | *Fatima* | *Wilma* | *Sabrina* | *WHAM* | *En/Spm* | *Sukkula* | *Cassandra* |
| G-13 | S1 | 1 | spikes | 52000 | 51100 | 53500 | 20900 | 30400 | 11100 | 2400 | 15700 | 7700 |
|  |  |  | leaves | 29400 | 28700 | 35200 | 14500 | 15600 | 6500 | 1700 | 10900 | 4300 |
|  | S2 | 1 | spikes | 49900 | 45000 | 53000 | 22400 | 31700 | 11700 | 2200 | 15500 | 8400 |
|  |  |  | leaves | 29500 | 28800 | 37500 | 15600 | 18500 | 7700 | 1700 | 11400 | 5500 |
|  |  | 2 | spikes | 54300 | 41100 | 48800 | 19800 | 23300 | 9900 | 2800 | 20000 | 8400 |
|  |  |  | leaves | 45200 | 38600 | 45700 | 19500 | 26100 | 9800 | 2200 | 15100 | 7600 |
|  |  | 3 | spikes | 54900 | 54600 | 46900 | 19800 | 33700 | 11900 | 3000 | 17300 | 9100 |
|  |  |  | leaves | 44500 | 64800 | 60500 | 17800 | 21800 | 9100 | 3500 | 17400 | 9000 |
|  |  | 4 | spikes | 58200 | 69300 | 71900 | 21300 | 26000 | 10400 | 3400 | 17500 | 9700 |
|  |  |  | leaves | 29500 | 32700 | 40700 | 15200 | 16300 | 7600 | 2100 | 13900 | 6000 |
|  |  | 5 | spikes | 37200 | 60100 | 54800 | 15400 | 17000 | 8200 | 3200 | 18900 | 8800 |
|  |  |  | leaves | 30000 | 35500 | 36100 | 13900 | 18300 | 8100 | 2100 | 12700 | 6000 |
|  | S3 | 1 | spikes | 50500 | 36800 | 49600 | 24100 | 34400 | 12800 | 2800 | 16800 | 8100 |
|  |  |  | leaves | 37800 | 33400 | 45100 | 19500 | 24600 | 9600 | 2300 | 12600 | 6800 |
| G-9 | S1 | 1 | spikes | 48700 | 32800 | 61000 | 24400 | 29900 | 12000 | 2400 | 14400 | 7300 |
|  |  |  | leaves | 34800 | 24100 | 44700 | 16400 | 21000 | 8300 | 2100 | 13400 | 5400 |
|  |  | 2 | spikes | 41100 | 27000 | 46300 | 22200 | 27800 | 11100 | 1900 | 13800 | 6000 |
|  |  |  | leaves | 27700 | 19500 | 30700 | 15000 | 16200 | 6700 | 1400 | 9200 | 4100 |
|  |  | 3 | spikes | 35800 | 77200 | 63800 | 25200 | 31100 | 12300 | 4400 | 16200 | 4200 |
|  |  |  | leaves | - | - |  | - | - | - | - |  | - |
|  | S2 | 1 | spikes | 46700 | 33200 | 57500 | 27100 | 30500 | 12100 | 2500 | 14700 | 7400 |
|  |  |  | leaves | 26300 | 17800 | 27900 | 13300 | 15000 | 6000 | 1300 | 8000 | 3500 |
|  |  | 2 | spikes | 52800 | 27800 | 57000 | 25600 | 29900 | 11500 | 2300 | 14400 | 6800 |
|  |  |  | leaves | 29400 | 21500 | 40000 | 15200 | 16500 | 6600 | 1700 | 9100 | 4600 |
|  |  | 3 | spikes | 53200 | 32200 | 69900 | 25800 | 34000 | 13200 | 2600 | 14900 | 7200 |
|  |  |  | leaves | 26800 | 21600 | 37200 | 15000 | 18500 | 7600 | 1900 | 10400 | 4700 |
|  | S3 | 1 | spikes | 51200 | 38300 | 59000 | 21800 | 25800 | 10800 | 2800 | 15500 | 7200 |
|  |  |  | leaves | 38100 | 41900 | 57600 | 15600 | 18500 | 7500 | 2600 | 15300 | 6500 |
|  |  | 2 | spikes | 38400 | 23700 | 45100 | 20700 | 22000 | 9200 | 1800 | 10800 | 5500 |
|  |  |  | leaves | 41000 | 37100 | 52100 | 18000 | 19200 | 8400 | 2300 | 11700 | 5900 |

## Table 2. Sequences with new TE insertion.

| TE | Sequence flanking new TE insertion (LTR termini in upper case) | Insertion into sequence |
| --- | --- | --- |
| ***Daniela*** | tacccctactttagtacaccgaca  ▼ atcttggtgatgatgcccttgagcagctgatcaagagcaaagaagaagcagaaatcttcagcaacctgcctctctttggtgtggccatcattcgcaatttcattgatgaatggttcgacacccccaacgtcagctttcaatatttacagctcccaattggcctcagcatcgccttcaatggcgccattgcttctgagctagctctcgctcagcgcattgttgagctcaaaaacaagattgattttgagaaggctcaattcaagaagcatgtggccaatctgagtgttcaagacgtccgcaacttcaaggtcatgctccatgagctcaaagaagcttttctgcagaaacgccaagaagctcagggctctcgagaacgcatgaagcttttggctgataagtgtgtgcttgcctacaatgaggctgagaagcgcaagtcccttggccgccctggtattgatcccaggatggctgcaaagaggaagaagaagcaccttgctgaacaggctgagccttcaagccaagaagcccctcgcattatcttcccgagcagcatgactggctcgaagcctaaggtcaccacaaccgcttcagaatagaagaagacaaaggctgctgctgctgctgct | EF081027  EF081025  AY951944  AF459639 |
| ***WIS2*** | TTTAATTTCTGCAACGTTCCCCAACA  ▼  gttatgttcgttgctcgcatgctacggccgagacatacagagaaagagagaggaagaagttaggtgtgtgagaaagatggagacatgtaatatatgtgagatgcgtgtgcatccggattctgtatacgtggaagaagaagagacaacatgtttgatgagagagctggaaaaacatggacgagaggggaaggatctcatgggttgagggattgacaattttgtgcgagggagagagggattggtaattttgtgcaagagagagagggtggggggaggagtcagtcaaccgtcgtcacattatcctacttccaaatgaccccgcattctctagcgcggtgtggtttccgtcttcgatctgctcgatgccctctttgaagattgaggttttgtgcttgttgtgctgcaaagaagcacaagcgagagtagtctccgtataaccttggatggggatgaattgtgtgctcaggggtgtgtgtgtgtgtgtgtgt | CT009588  CT009585  DQ138092 |
| ***Sukkula*** | TGATGAATCGCATCTTGGGCGTGACA  ▼  ttggtgcccctgggaactgtgatataaaaccactctcgttgccataggccgagctccttttgaaaagagccctcgggccacggagcatcggcatccttgcttataacagcccctccgcactctgcctgctgccccttgatcatcttcggctccaccttgaaggtcttgagccacaatccgaagtgaggggtaatgcagaggaaggcttcgcatatgatgatgaacgatgagatctggaggatggactccggatccaagtcatgaaattccagcccataataaaacatgagccccctcacgaacgggtccgtcgggaagcctagcccccgaagaaagtgagacgcgaacaccacgctttcgccaggcttgggagtgggaataacctgcccttgggcaggcagcctatgcgaaatttcaccggttagatacttggcatctcttagctttagcacgtcttcttccgtaacggaggaaggcacccaccggccttgcaggtcggagccggacatcattgaaggtctgaagcacctgaatctggagctttggatgttggaactcgaggcgaggggcggattcgattgagattgaaagaaaaaagtggagccttggtctctttataaagaggttgaataccaagagccttccccatgaccgttcgggactcgccttcgatggatgggacgtaccaacaggcacgattgggttacccatgcccgtattgatgagaatcctggaataaggggacacgatctctgctttgacaagacgtgccaaggaaaccgccttgctaaacgcgctaaggtgggacagtaaaaacgattagaataaaggcttggccgtggtgtgatgtcacgccaaagatgcgattctata | EF426565  AY146587 |
| ***Sukkula*** | ATGAAGATCGCATCTTGGGCGTGACA  ▼  gtagtagaacatgagcccccggacaaatgggtgaagaggaaaacctagtccgcagaggaagtgggtgaggagcaccaccctctcatggggcccgggggtggtgatgagctgccccttgtcggggagccgatgcgcgatgtcgctggacaagtatccggccttttgcagcctcttgatgtgcccatccatgacggaggaggccatccacttgtctcctgctccggacatggctggggaaggttgaggcgagatgagcggacttgggagctggagctcgagtgcgcggaaatggataagcaaaggaggaagaaggaataggtaaaaaggtggatccttatccccttatatggacggacgaaactatgcgtccccaccagcctagtaaaactcgcttatctcccaagcccccctaatcaaatggcgcggttgggtacccccgcccgtatttataccaatctcgaaataatgggacacacatctttgtttgctttgacatgacaggccaccacatcgacatcgtgctgtggggtgaggtgaaaacgaatcaaataattgcttggacggggttgtgatgtggtgtcatgacgcgattctatatatcaattctatcagcgcctgctgggggccccaaaaag | DQ537335  AY494981  AY663391  EF179137  EF179138  AF326781 |

## Table 3. Transposable element accessions.

| TE, Superfamily | Accessions |
| --- | --- |
| *WIS2, Copia* | TREP1723, TREP1724, TREP839, TREP840, TREP841, TREP262, TREP1823, TREP1824, TREP1825, TREP1826, TREP818, TREP819, TREP1325, TREP1439, TREP1440, TREP1441, TREP1442, TREP1443, TREP10, TREP96,TREP105 |
| *Daniela, Gypsy* | TREP796, TREP1226, TREP2208, TREP231, TREP1408, TREP1228 |
| *Fatima, Gypsy* | TREP827, TREP828, TREP252, TREP1229, TREP1230, TREP1804, TREP2209, TREP1231, TREP1232, TREP1306, TREP1413, TREP1414, TREP1415 |
| *Wilma, Gypsy* | TREP842, TREP820, TREP821, TREP822, TREP1438, TREP2210 |
| *Sabrina, Gypsy* | TREP260, TREP708, TREP709, TREP812, TREP2218, TREP710, TREP736, TREP1430, TREP1431, TREP1432, TREP1239, TREP259 |
| *WHAM, Gypsy* | TREP263, TREP264, TREP265, TREP1243, TREP815, TREP817, TREP2224, TREP1244, TREP49, TREP1320, TREP1321, TREP109 |
| *En/Spm, CACTA* | AY265311, AY707998, TAE406397, AY265312, AY708002, TAE406398, AY708004, AY707999, TAE406396, AY707996, AY708006, AY708003, AY708005, AY772017, AY708001, AY707997 |
| *Sukkula (LARD), unknown* | TREP102, TREP1213, TREP1214, TREP1215, TREP1216, TREP1217, TREP1240, TREP1314, TREP208, TREP209, TREP258, TREP715, TREP740, TREP1550, TREP1551, TREP1552, TREP1553, TREP1554, TREP1555, TREP1556, TREP1648, TREP1649, TREP2038 |
| *Cassandra* (TRIM), *unknown* | AY271963 |
| *Spelt52* (non-TE) | Z21644, AY117400, AY117401, AY082346, AY082347 |

## Table 4. Specific primers and primer sets for *Aegilops* TEs, 5S rRNA genes and *Spelt52* tandem repeats.

| Oligo name | Sequence | Nucleotide  Position in  accession | Identity | PCR  product size, nt |
| --- | --- | --- | --- | --- |
| 1607  1608 | tgaaggaaatatgccctagaggc  tcgaccatttcgagactcctcgt | TREP1219:  6->28  926<-948 | *WIS2* LTR | 945 |
| 1609  1610 | tgagtatgacaaccggcaggag  agtcaaccccttgaacttgtcg | TREP1724:  2263->2284  5561<-5582 | *WIS2* internal domain | 3400 |
| 1603  1604  1700 | tggtatcagagcctcgaccgac  tccaccactacgagaggatctcg gttatgcaatctggtgttgggga | TREP258:  5538->5559  9095<-9117  6141<-6163 | *Sukkula* internal domain | 4500 (1603-1604)  610 (1700-1603) |
| 1605  1606 | tgtcacgcccaatatgcgacc  tgtaacgccccggatacaac | AF453665:  4305<-4325  TREP208:  1->21 | *Sukkula* LTR | 4000 |
| 873  874 | atatcttgtgcatcgggattcc  gacataaccccaccgtgtcctc | AF453665:  3393->3415  3691<-3713 | *Sukkula* LTR | 320 |
| 1615  1617 | tgaagaggaaagggtgatgcagc  gcaggaattaagcttggggatgc | TREP260:  7470<-7492  5927->5949 | *Sabrina* LTR | 1500 |
| 1618  1620 | acgctactttgctgcatcacc  agcttaattcctgctcgtcctcga | TREP260:  1508->1528  5917<-5940 | *Sabrina* internal domain | 3600-5500 |
| 1621  1622 | tgaatggaactaacccggactga  acccacaagtataggggatcgca | TREP263:  71->93  1368<-1390 | *Wham* LTR | 1350 |
| 1624  1625 | ggaactgccatctagctctgcac  aacccttgcttgtcctcaagc | TREP263: 1502->1524  8103<-8123 | *Wham* internal domain | 6600 |
| 1635  1636 | agcgaggtctaagcttgggggag  gatgacccacaagtataggggatc | TREP821:  11374->11396  12857<-12880 | *Wilma*/*Bagy2* LTR | 1508 |
| 1637  1638 | agatcccctatacttgtgggtc  tcccccaagcttagacctcgct | TREP821:  1402->1423  11374<-11395 | *Wilma* internal domain | 7200-10000 |
| 1694  1695 | tcagaaaggatttgcaggtgc tgaacagccaaatgcaccatcac | 56->76  460<-482 | *En*/Spm | 428 |
| 1696  1697 | cgatcaggacgacgccaaatcg catcttcggtctccaggcgga | TREP231:  6966->6987  7335<-7355 | *Daniela* internal domain | 391 |
| 1698  1699 | gggactttccttggcgtggaagg tcaaaaccggcggatctcgggt | TREP1804:  108->130  454<-475 | *Fatima* LTR | 369 |
| 784  977 | cgagtgaggacaaagtgcgcag ttgtcctcactcatgcgcacc | AY271963:  161->182  613<-633 | *Cassandra* whole element | 474 |
| 621  620 | ctggagcaattttaggatgggtgacc  tgctctcgcccaagcacgcttaac | 67->92  51<-74 | 5SrRNA with spacer | 455 |
| 1803  1804 | ggatgcgatcataccagcact  gggaatgcaacacgaggacttc | 1->21  99<-120 | 5SrRNA gene | 120 |
| 1760  1761 | cttctctagcctgagcctgacac  gttccgggaaatctcggcccaaa | Z21644:  313<-335, 701<-723;  150->172, 539->561 | *Spelt52* | 186 |
| 1762  1763 | agaatagtgtgtcaggctcagg  ccaaatggatctacggcaccatcg | Z21644:  305->326, 693->714;  246<-269,  634<-657 | *Spelt52* | 354 |

## Table 5. Primer combinations for quantitative PCR

| TE, region | Primer combination, size of PCR product | First detection of fluorescence (Ct), average error |
| --- | --- | --- |
| *WIS2*, LTR | 1607 and 1608, 945bp | 4 cycles, 6.0% |
| *Daniela*, internal domain | 1696 and 1697, 391bp | 10 cycles, 6.0% |
| *Sabrina*, LTR | 1615 and 1617, 1500bp | 10 cycles, 8.1% |
| *Wham*, LTR | 1621 and 1622, 1350bp | 14 cycles, 4.9% |
| *Wilma*, LTR | 1635 and 1636, 1508bp | 9 cycles, 5.9% |
| *En*/*Spm* | 1694 and 1695, 428bp | 12 cycles, 12.8% |
| *Sukkula*, internal domain | 1603 and 1700, 610bp | 10 cycles, 5.0% |
| *Sukkula*, LTR | 1605 and 1606, 4300bp | 18 cycles, 18.0% |
| *Fatima*, LTR | 1698 and 1699, 369bp | 8 cycles, 4.7% |
| *Cassandra*, LTR and internal domain | 784 and 977, 474bp | 17 cycles, 5.8% |
| 5SrRNA and spacer | 620 and 621, 455bp | 13 cycles, 7.0% |
| *En*/*Spm* | 1694 and 1695, 428bp | 14 cycles, 4.2% |
| *Spelt52* (non-TE) | 1762 and 1763, 436bp | 7 cycles, 5.9% |
| *Spelt52* (non-TE) | 1760 and 1761, 576bp | 10 cycles, 10% |

## Table 6. Primers for IRAP analysis.

| **Name** | **Sequence** | **TE, region** |
| --- | --- | --- |
| 560 | ttgcctctagggcatatttccaaca | *Wis2*, LTR |
| 554 | ccaactagaggcttgctagggac |  |
| 2105 | actccatagatggatcttggtga |  |
| 2106 | taatttctgcaacgttccccaaca |  |
| 2107 | agcatgatgcaaaatggacgtatca | *Wilma*, LTR |
| 833 | tgatcccctacacttgtgggtca |  |
| 2108 | agagccttctgctcctcgttgggt |  |
| 516 | tcctcgttgggatcgacactcc |  |
| 2109 | tacccctactttagtacaccgaca | *Daniela*, LTR |
| 2110 | tcgctgcgactgcccgtgcaca |  |
| 2111 | caggagtagggttttacgcatcc |  |
| 2112 | tgctgcgactgcccgtgcaca |  |
| 2113 | tacgcatccgtgcggcccgaac |  |
| 2114 | ggacaccccctaatccaggactcc | *Fatima*, LTR |
| 2115 | caagcttgccttccacgccaag |  |
| 2116 | cgaacctgggtaaaacttcgtgtc |  |
| 2117 | agatccgccggttttgacaccgaca |  |
| 728 | tgtcacgtccaagatgcgactctatc | *Sabrina*, LTR |
| 2118 | gtagataatatagcatggagcaatc |  |
| 2119 | agccactagtgaaacctatgg |  |
| 2120 | gtgacctcgaagggattgacaacc |  |
| 2121 | actggattgataccttggttctcaa |  |
| 2122 | agggaaatacttacgctactctgc |  |
| 58 | tgatgaatcgcatcttgggcgtgac | Sukkula, LTR |
| 65 | atgaagatcgcatcttgggcgtgac |  |
| 2123 | ggaaaagtagatacgacggagacgt | Wham, LTR |
| 483 | tctgctgaaaacaacgtcagtcc |  |
| 1623 | tgcgatcccctatacttgtgggt |  |
| 2124 | ccttcaatattaagaagtgtgccga | En/Spm |
| 2125 | ggcactacttagaggtccagta |  |

## Table 7 The significance of deviations in each generation via Ttest and non-parametric Mann-Whitney Utest

| **Tissue** | **Element** | **avR** | **avS** | **General**  **SD** | **dfR** | **dfS** | **Ttest** | **Tt_pval** | **Ur** | **Us** | **mU** | **sdU** | **zU_pval** | **si** | **Tt2m** | **Tt2p** | **Ftest** | **Fpval** | **Log**  **Fpval** |
| --- | --- | --- | --- | --- | --- | --- | --- | --- | --- | --- | --- | --- | --- | --- | --- | --- | --- | --- | --- |
| leaves | Cassandra | 4369,5 | 7068,92 | 162,793 | 2 | 100 | -16,5819 | **9,26E-31** | 0 | 200 | 100 | 41,43268 | **0,007899** | -1 | 274,9594 | 1,446167 | 48,31604 | 0,004872 | **-5,32425** |
| leaves | Daniela | 29742 | 37990,1 | 1426,6 | 2 | 100 | -5,78164 | **4,23E-08** | 0 | 200 | 100 | 41,43268 | **0,007899** | -1 | 33,42736 | 0,902107 | 4,356659 | 0,128988 | **-2,04804** |
| leaves | Fatima | 32477,5 | 43051,9 | 971,321 | 2 | 100 | -10,8866 | **5,69E-19** | 0 | 200 | 100 | 41,43268 | **0,007899** | -1 | 118,5181 | 1,467011 | 22,57922 | 0,014637 | **-4,22421** |
| leaves | Sabrina | 14305,5 | 29895,6 | 731,378 | 2 | 100 | -21,3161 | **2,83E-39** | 0 | 200 | 100 | 41,43268 | **0,007899** | -1 | 454,3761 | 0,038622 | 158,3495 | 0,000842 | **-7,07929** |
| leaves | Sukkula | 13038 | 19108,6 | 638,54 | 2 | 100 | -9,50704 | **5,96E-16** | 0 | 200 | 100 | 41,43268 | **0,007899** | -1 | 90,38381 | 2,379892 | 41,74727 | 0,006031 | **-5,11078** |
| leaves | WHAM | 7005 | 12069,8 | 283 | 2 | 100 | -17,8968 | **3,02E-33** | 0 | 200 | 100 | 41,43268 | **0,007899** | -1 | 320,2955 | 2,449 | 219,3358 | 0,000518 | **-7,56485** |
| leaves | Wilma | 12889 | 28200,9 | 908,327 | 2 | 100 | -16,8573 | **2,74E-31** | 0 | 200 | 100 | 41,43268 | **0,007899** | -1 | 284,1686 | 1,302805 | 431,553 | 0,000189 | **-8,576** |
| leaves | WIS2 | 29738,5 | 48143,5 | 1173,6 | 2 | 100 | -15,6825 | **5,27E-29** | 0 | 200 | 100 | 41,43268 | **0,007899** | -1 | 245,9408 | 3,258983 | 65,54473 | 0,003113 | **-5,7721** |
| spikes | Cassandra | 7498 | 7068,92 | 163,328 | 2 | 100 | 2,62711 | **0,004983** | 186 | 14 | 100 | 41,43268 | **0,018963** | 1 | 1,24081 | 6,901707 | 45,84471 | 0,005261 | **5,24746** |
| spikes | Daniela | 46396,5 | 37990,1 | 1498,52 | 2 | 100 | 5,60982 | **9,04E-08** | 194 | 6 | 100 | 41,43268 | **0,011642** | 1 | 1,616495 | 31,47008 | 31,55523 | 0,009055 | **4,70447** |
| spikes | Fatima | 47452,5 | 43051,9 | 1112,01 | 2 | 100 | 3,95735 | **7,09E-05** | 109 | 91 | 100 | 41,43268 | **0,414019** | 1 | 1,435339 | 15,66062 | 47,10078 | 0,005057 | **5,286981** |
| spikes | Sabrina | 28767,5 | 29895,6 | 736,824 | 2 | 100 | -1,53106 | **0,064456** | 74 | 126 | 100 | 41,43268 | **0,265158** | -1 | 2,344145 | 0,597282 | 11,0157 | 0,039726 | **-3,22575** |
| spikes | Sukkula | 15614 | 19108,6 | 601,06 | 2 | 100 | -5,8141 | **3,66E-08** | 0 | 200 | 100 | 41,43268 | **0,007899** | -1 | 33,80376 | 2,008224 | 23,98732 | 0,013427 | **-4,31047** |
| spikes | WHAM | 11012 | 12069,8 | 278,967 | 2 | 100 | -3,79181 | **0,000128** | 2 | 198 | 100 | 41,43268 | **0,009008** | -1 | 14,37782 | 1,683817 | 18,01502 | 0,020148 | **-3,90465** |
| spikes | Wilma | 21260,5 | 28200,9 | 893,631 | 2 | 100 | -7,76652 | **3,6E-12** | 0 | 200 | 100 | 41,43268 | **0,007899** | -1 | 60,31883 | 0,172384 | 305,9568 | 0,000315 | **-8,06179** |
| spikes | WIS2 | 50045,5 | 48143,5 | 1191,95 | 2 | 100 | 1,59571 | **0,056855** | 142 | 58 | 100 | 41,43268 | **0,155365** | 1 | 0,430403 | 2,54629 | 2,094922 | 0,279567 | **1,274513** |
